# A hybrid theoretical–numerical–experimental framework for robust health monitoring of thin-walled hollow composite members using guided waves

**DOI:** 10.1038/s41598-025-96150-z

**Published:** 2025-04-09

**Authors:** Akshay Prakash Kalgutkar, Shirsendu Sikdar, Sauvik Banerjee, Karl Walton, Rakesh Mishra

**Affiliations:** 1 Civil Engineering Department, Indian Institute of Technology Bombay, Powai, Mumbai, 400076 India; 2 School of Computing and Engineering, University of Huddersfield, Queensgate, Huddersfield, HD1 3DH UK

**Keywords:** Hollow composite member, Guided wave, Surface abrasion, Hairline crack, Piezoelectric transducers, Engineering, Aerospace engineering, Energy infrastructure, Mechanical engineering

## Abstract

Thin-walled hollow composite members (HCM) are extensively employed in aerospace and automotive industries due to their high strength-to-weight ratio and design flexibility. This study introduces a hybrid -numerical–experimental framework for robust detection and characterisation of barely visible damage in HCM using guided waves (GW). It focuses on assessing surface abrasion and hairline cracks, two common yet challenging damage types encountered in the field. A semi-analytical finite element (SAFE) formulation is developed for the dispersion analysis alongside numerical simulations using finite element software COMSOL Multiphysics^®^, and experimental validation is performed to ensure accurate and reliable results. The study focuses on GW propagation and scattering behaviour under varying damage scenarios, exploring the effects of damage size, position, and its offset on wave features. Parametric analyses show significant variations in wave characteristics such as group velocity, amplitude, and mode features. A waveform and statistical approach incorporating continuous wavelet transform (CWT) and energy enables precise damage classification. Results show that abrasion-induced damages cause substantial changes in GW features in terms of DIs and statistical parameters, while hairline cracks marginally affect the damage indices and wave features, aiding in distinguishing between different damage types. These findings contribute to the development of robust damage identification algorithms for structural health monitoring, providing valuable insights for optimising the maintenance and performance of composite structures in critical engineering environments, ensuring safety and operational efficiency.

## Introduction

Thin-walled hollow sections are widely utilised across various engineering domains due to their remarkable strength-to-weight ratio. Among these, square hollow sections stand out for their enhanced resistance to torsion and bending, making them the preferred choices for structural components in buildings, machinery, and mechanical systems^1–4^. Meanwhile, fibre-reinforced composite materials are a special type of engineering material that finds its applicability in most of the engineering field due to their versatility and exceptional mechanical properties like high strength-to-weight ratio, durability, and resistance to corrosion and chemicals. When fabricated from these composites, square hollow sections become adaptable structures for advanced engineering uses. However, these structures are susceptible to various forms of damage, such as hairline cracks, surface wear or abrasion damage, fibre breakage, debonding, and other defects due to cyclic loading, impacts, ageing, and mishandling. If these issues remain undetected and unaddressed in the preliminary stages, they can escalate to cause severe structural failures after compromising the structure’s residual strength^5–8^. These issues are exacerbated by the frequent use of these structures in concealed locations, where conventional inspection methods are ineffective. As a result, regular inspections using suitable damage detection methods are crucial to maintain the structural integrity and safety of a building or system. Consequently, the need for Non-Destructive Evaluation (NDE) and Structural Health Monitoring (SHM), specifically for square hollow sections, is increasing rapidly and holds considerable importance in designing damage-tolerant structures and for maintaining the safety and performance of such structures. Further, SHM can also help improve the design of advanced structures by providing more accurate information about failure modes and the loads on the structures.

Damage assessment in composite structures poses significant challenges due to their anisotropic nature^9–11^ and the varying arrangement of plies. Consequently, assessing the health of composite materials necessitates specialised techniques. Despite these challenges, numerous non-destructive testing (NDT) methods exist for detecting and characterising both surface and internal structural discontinuities without compromising structural integrity. Among these, ultrasonic-guided wave (GW) methods stand out for their efficiency and accuracy in monitoring damages or discontinuities in composite structures. These methods are particularly promising due to their capability for long-range monitoring and their sensitivity to minor defects, making them ideal for both localising and characterising hidden structural issues^12,13^. The current SHM strategies typically involve the use of cost-effective, thin, and lightweight bonded piezoelectric or broadband transducers^5,14^, emphasising the importance of understanding the dispersive behaviour of Lamb waves for successful SHM of composites using piezoelectric transducers^5,15^.

Lamb wave-based damage detection methods have been widely applied in inspecting thin structures^16–20^. However, their implementation in composite panels is complicated due to the complex nature of Lamb wave propagation, characterised by multi-mode features, dispersion, and direction dependence of wave velocity^21,22^. To tackle the challenges posed by complex mode conversion due to changes in waveguide thickness, Younho^23^ introduced a hybrid boundary element method for analysing Lamb waves, enabling better management of wave mode conversion in varying thicknesses. Further, few researchers have explored the effect of surface abrasion or corrosion on the guided wave interaction characteristics in the isotropic and laminated thin structure. Banerjee and Kundu^24^ proposed a novel semi-analytical technique known as the Distributed Point Source Method (DPSM) for modelling the ultrasonic field in sinusoidally corrugated waveguides immersed in water, while Lee et al.^25^ demonstrated the use of non-contact laser ultrasonic propagation imaging for detecting cracks and surface corrosion in aluminum plates. Their approach utilised an Nd: YAG pulse laser system for wave generation and a galvanometer-based laser scanner for initial area scanning. Similarly, Wang et al.^26^ investigated the effect of surface abrasion in the composite plate on the guided wave response, employing a Gaussian mixture model (GMM) for damage localisation. De Luca et al.^27^ explored the characterisation of low-velocity impact damage in composite plates using guided waves. In contrast, Hua et al.^28^ proposed a time–frequency damage index derived from broadband Lamb wave for corrosion inspection in aluminium plates. Tavaf and Banerjee^29^ developed a generalised Rayleigh–Lamb equation for corrugated waveguides with a single equation employed for both flat waveguides and corrugated and tapered plates, facilitating a physics-based predictive design of wave filters. Further, a nonlinear-based wave propagation investigation was conducted by Sikdar et al.^30^ on a stiffened composite plate with baseplate-stiffener breathing debonding by a mean of numerical approach and experiments, proposing a baseline-free online breathing-debond source localisation strategy based on the changes in second harmonic response magnitudes. Later, Sha et al.^31^ proposed a guided wavefield curvature technique for mapping invisible structural damage in composite structures by fusing the energy images. Recently, Ahmed et al.^32^ used coda wave interferometry (CWI) and nonlinear ultrasonics (NLU) for the quantification of precursor damage in carbon fiber-reinforced polymer (CFRP) composite structures.

Beyond inspecting thin-walled structures, ultrasonic-guided wave techniques have been successfully applied for the evaluation of various other structural systems like railways^33–35^ and steel strands^36,37^. Circular pipelines have also garnered significant research attention^38,39^. For instance, Wan et al.^40^ carried out the guided wave inspection to inspect the hexagonal pipe for the purpose of detecting the damage. They employed a semi-analytical finite element (SAFE) model to develop the dispersion curve and subsequently conducted numerical analyses of wave propagation using Abaqus CAE. Wave propagation of non-axisymmetric guided waves in a hollow cylinder was conducted by Li and Rose^41^ through the normal mode expansion method (NME). Their study incorporated various sources such as angle beam, tube end excitation with normal beam, and comb transducer and found that the phase velocities for different modes vary with propagating distances. Later, Barshinger and Rose^42^ examined the propagation of ultrasonic-guided waves in an elastic hollow cylinder with a viscoelastic coating through the global matrix technique to derive the dispersion equation for the longitudinal modes. Subsequently, Sanderson et al.^43^ introduced an analytical model to investigate the scattered wave response in the pipe bends. Through this analytical model, they could predict both the effect of pipe bends over a long range and the distortion caused by pipe bends. Later, Guan et al.^44^ devised an effective approach for detecting microcracks resulting from fatigue in plate and pipe structures using nonlinear guided waves. Yeung and Ng^45^ utilised time domain spectral finite element to study the guided wave effect in the aluminium pipe. By leveraging the torsional mode, they explored the interaction of the guided wave with crack through mode conversion effect, which aids in the damage detection in the pipe.

Despite advancements in guided wave research, limited studies focus on ultrasonic wave propagation in square hollow sections. Houillon et al.^46^ examined the wave propagation characteristics in an isotropic thin-walled structure with any cross-section by developing a dispersive curve for both hollow circular pipes and square hollow sections using a finite element method. The damage detection in the concrete-filled square steel tube through the embedded piezoceramics transducer by using the ultrasonic method was carried out by Zhang et al.^47^. Subsequently, Gao et al.^48^ employed traditional ultrasonic testing by exciting specific longitudinal waves to detect delamination in a CFRP square tubular section. On the other side, Wan et al.^49^ carried out experimental and numerical ultrasonic-guided wave inspections on steel square tubes to study wave interactions with circular through-hole damages on surfaces and slot damages at edges using SAFE-derived dispersion curves and finite element simulations. Yang et al.^54^ recently proposed an ultrasonic-guided wave-based damage detection method to detect discontinuities in a square steel tube using structure symmetry. They initially developed a dispersion curve using the SAFE method and then designed a symmetric layout scheme for transmitters and receivers based on the tube’s symmetry, proposing a signal processing strategy for damage detection. Later, Li et al.^50^ proposed a physics-informed deep filtering method to enhance the weak guided wave signal in the square steel tube. They constructed a deep learning model trained on guided wave signals obtained from numerical simulations, which was then used to enhance weak ultrasonic guided wave signals from real-world scenarios. By building on these studies, the current research aims to advance the understanding of guided wave propagation in thin-walled HCMs and develop robust techniques for detecting and characterising damage in these critical structural components.

### Research gap and scope of the present study

From the review of the existing literature, it is evident that substantial research has been conducted on wave propagation and damage detection in thin-walled composite plates and isotropic circular pipe sections. There is a significant gap in the study of damage detection in thin square hollow tube sections, despite extensive investigations into damage assessment in homogeneous isotropic structures using ultrasonic guided waves, particularly in flat and curved configurations. Little attention has been given to understanding the dispersion behaviour of various wave modes in thin-walled hollow composite members (HCM), especially through the development of dispersion curves. Moreover, a critical research gap exists in examining the sensitivity of the scattered guided wave response to different types of in-service damages or discontinuities in thin-walled hollow composite sections. Notably, damage scenarios involving crack formation and surface corrosion remain underexplored in the existing literature.

The current research aims to fill these gaps by investigating the GW propagation, dispersion, and interaction with different types of pragmatic in-service damages like ‘hairline cracks’ and ‘abrasions’ in thin-walled hollow composite members (HCM). To achieve this objective, numerical dispersion analysis of propagating guided waves is conducted using a semi-analytical finite element (SAFE) method and a 3D time-domain numerical model is developed in COMSOL Multiphysics^®^. The developed model is the multiphysics problem that couples the solid mechanics module with the electrostatics problem. In the current study, the two most commonly occurring in-service damage types are considered for the HCM: barely visible damage (abrasion) and hidden damage (hairline crack). The wave responses obtained from the performed laboratory experiments under different damage conditions serve to validate the developed FE model. Further, comprehensive parametric investigations are undertaken by taking into account the variations in the sizes and positions to evaluate the variations in wave responses through various wave features with respect to the different types of damage, thereby contributing to a deeper understanding of how these damages affect guided wave response. This research is expected to significantly advance the field of SHM for thin-walled hollow composite members, providing valuable insights for developing improved SHM strategies and damage detection methods for such structures in practical applications.

## Finite element simulation in COMSOL multiphysics

**Fig. 1:**
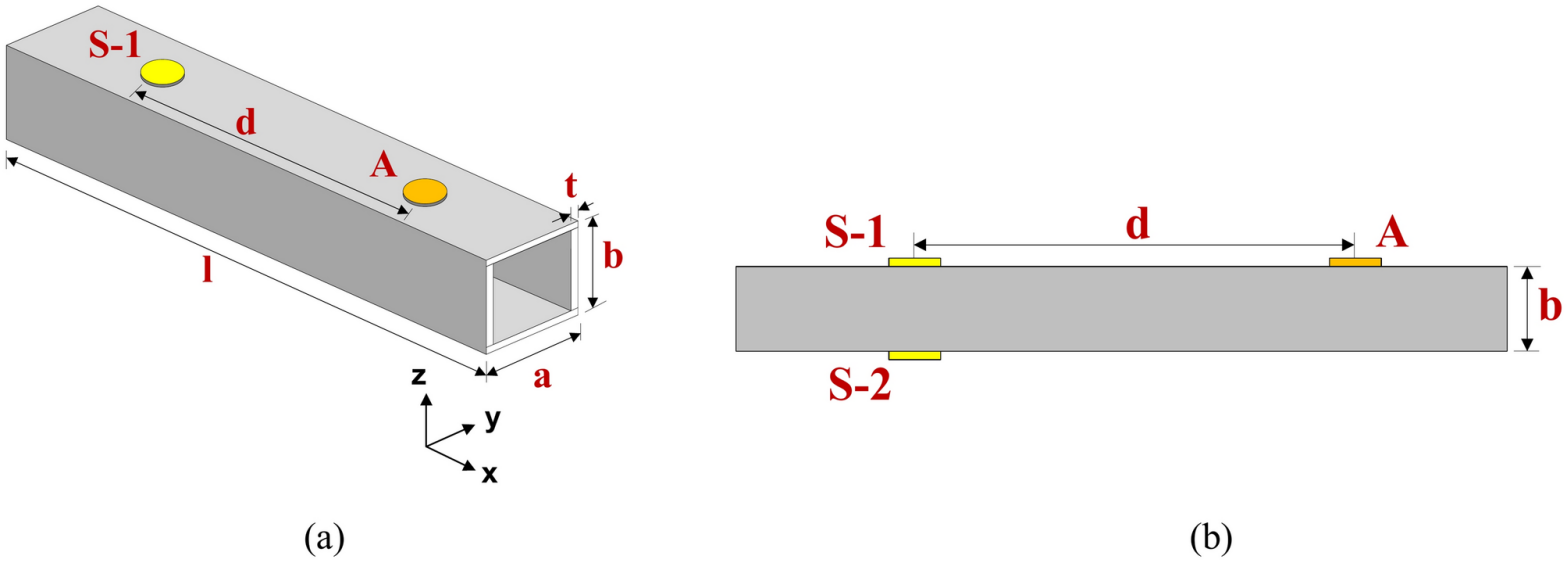
Model of HCM attached with the piezoelectric patches.

**Fig. 2:**
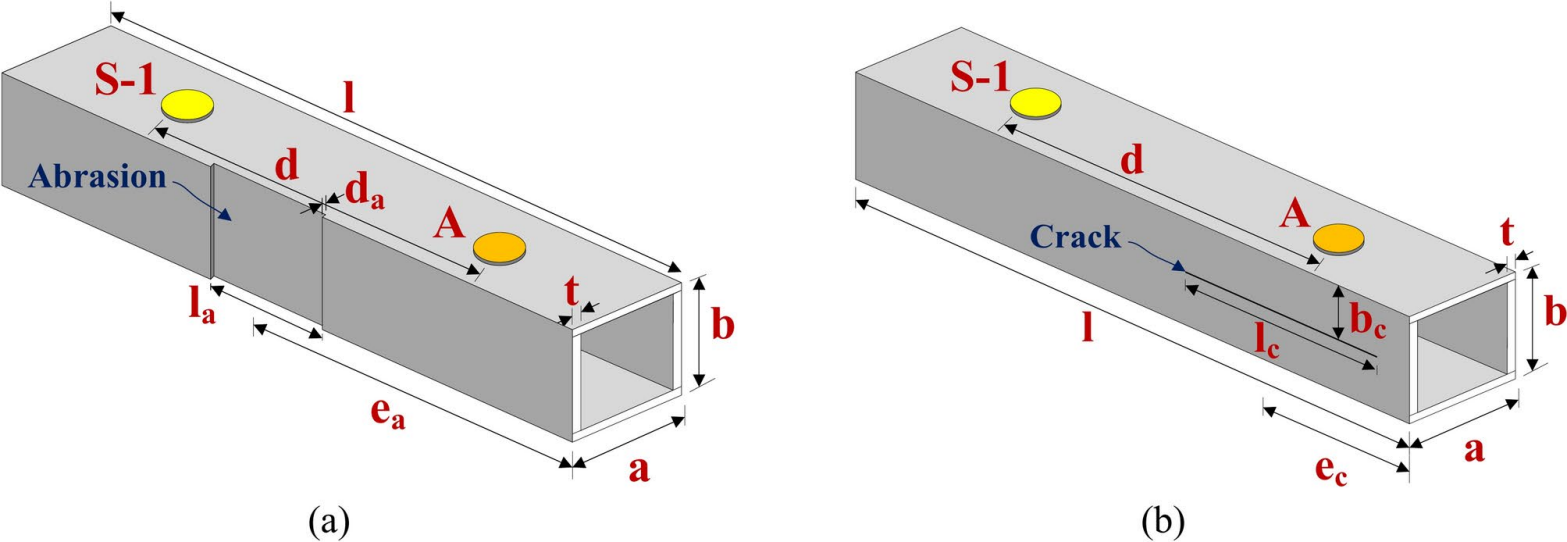
Model of HCM with various damages on the face of the section.

The current research aims to investigate wave propagation interaction with various damages in a HCM using circular PZT patches attached to the surface through finite element modelling. The analysis is performed using COMSOL Multiphysics version 5.5, focusing on a HCM with different types of surface damage, such as abrasion and hairline crack. The CFRP hollow composite section considered has a width and height of ‘*a*’ and ‘*b*’ and a length of ‘*l*’ in the y, z, and x directions, respectively. The wall thickness of the hollow section is ‘*t*’, as depicted in Fig. 1a. Additionally, three circular piezoelectric (PZT) patches, each with a diameter of 10 mm and a thickness of 0.4 mm, are attached to the surface of the hollow section, as illustrated in Fig. 1b. The section is excited using the PZT ‘A’, and the scattered response is captured through two PZT patches, ‘S-1’ and ‘S-2’ attached on the opposite surface of the section, at a distance ‘*d*’ from actuator PZT ‘A’. The study considers two types of damage, i.e., abrasion and hairline crack on the surface of the hollow section, as illustrated in Fig. 2. The abrasion considered is a through width damage with a depth of ‘*d*_*a*_’ and a length of ‘*l*_*a*_’ positioned at a distance of ‘*e*_*a*_’ from the edge closer to the actuator PZT ‘A’. The abrasion is modelled by removing a portion of uniform depth from the surface within the region of interest, as shown in Fig. 2a. On the other side, the hairline crack is considered on the face by creating a thin groove of 0.4 mm depth and 0.1 mm width with a length of ‘*l*_*c*_’ located at a distance ‘*b*_*c*_’ from the actuator attached surface and at a distance of ‘*e*_*c*_’ from the closer edge to the actuator PZT ‘A’, as presented in Fig. 2b. The PZTs are strategically positioned on the different faces of the damaged surface, enabling the assessment of inaccessible defects by attaching the sensor in the accessible zone in the field.

**Fig. 3:**
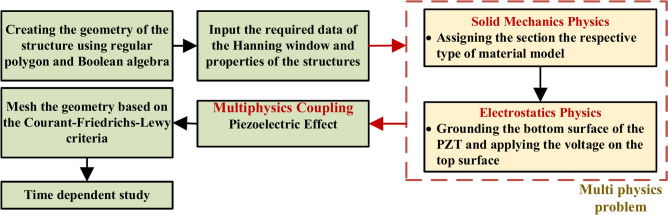
Flowchart of the simulation conducted in COMSOL Multiphysics® for GW propagation study.

This section outlines the physics involved and the initial steps utilised for the study in COMSOL Multiphysics®. The investigation considers the solid mechanics and electrostatics modules coupled with the multiphysics module termed the “piezoelectric effect”. Further, a time-dependent study is employed to simulate wave propagation in the HCM. Figure 3 presents a flowchart detailing the steps involved in simulating guided wave propagation in the hollow composite square section with various defects, which are elaborated upon in the subsequent section.

### Physics and solver involved in simulation

**Fig. 4:**
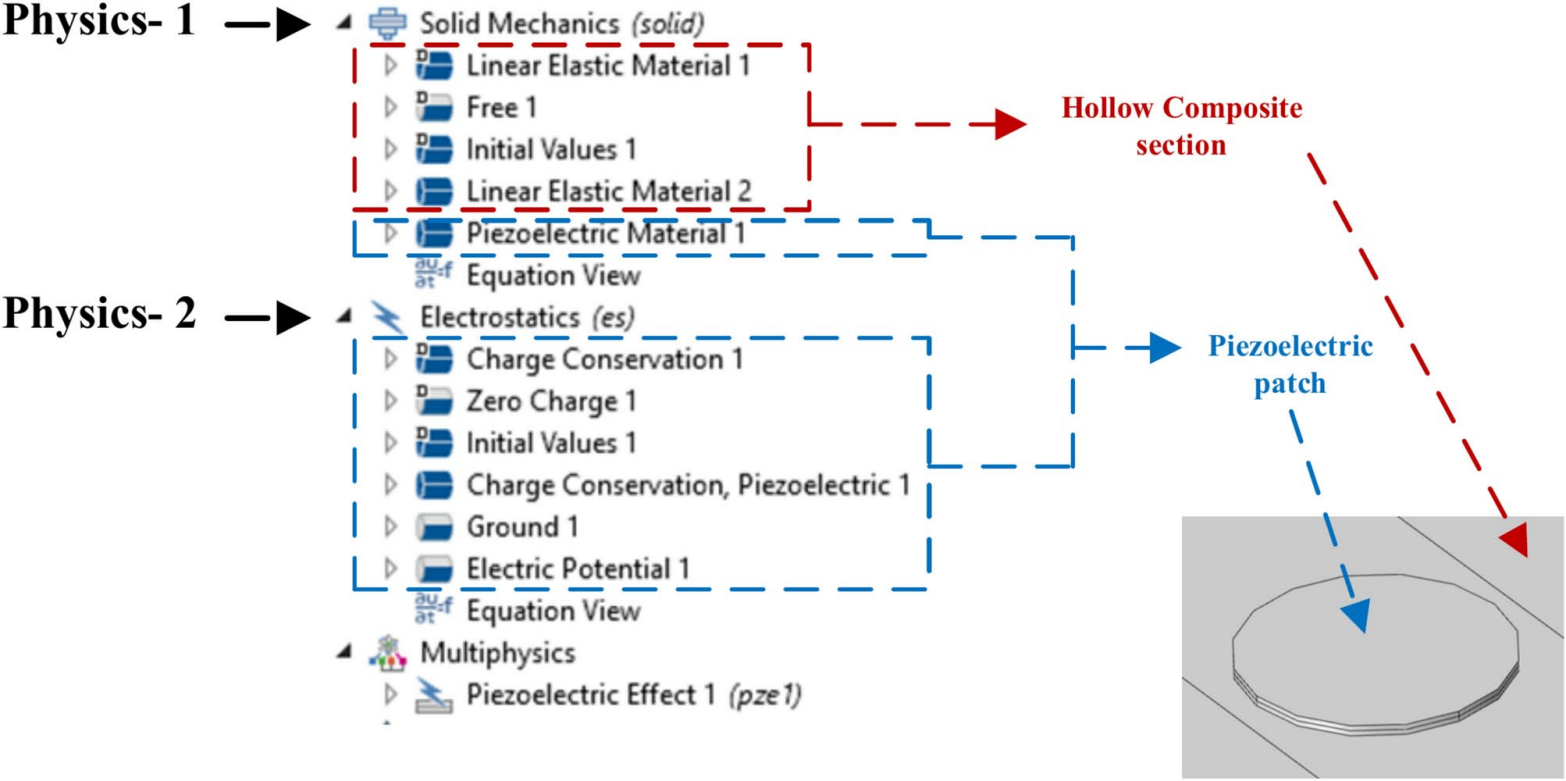
The simulation physics in COMSOL Multiphysics® for GW propagation in HCM.

The physics involved in simulating guided waves (GW) in HCM is illustrated in Fig. 4. A detailed description of the various steps executed in each physics module during the simulation is elaborately explained. In Solid Mechanics, the engineering properties are assigned to both hollow sections and piezoelectric patches by Linear Elastic material and Piezoelectric material options, respectively, to conduct the mechanical investigation. Further, due to the anisotropic nature of the composite material, the local material axes of the top and bottom face of the section are aligned along the global axes, while the local material y and z axes of the side faces are rotated by 90° with respect to global x- axes using the rotated system module, simulating the alignment of the woven fabrics along the length of the hollow specimen. This alignment is achieved by using two Linear Elastic Material options in the solid mechanics module, as presented in Fig. 4. In the electrostatics module, the electric constraints for the piezoelectric sensors are specified by grounding the lower surface of PZT patches to zero potential. Additionally, for the time-dependent study, the voltage waveform of the GW excitation is applied to the upper surface of the piezoelectric patch. Further, the Piezoelectric Effect Multiphysics module combines Solid Mechanics and Electrostatics modules to simulate the sensor’s piezoelectric behaviour.

**Fig. 5:**
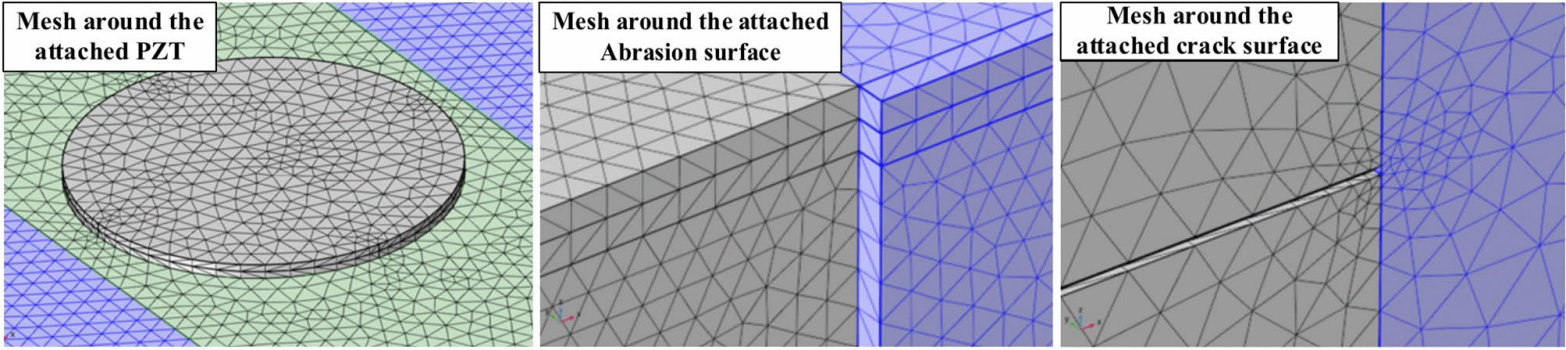
Tetrahedral mesh scheme around the PZT patch and damage edge.

A physics-controlled tetrahedral mesh is employed to discretise the developed model into smaller elements based on the involved physics, thereby ensuring effective convergence of the results. Previous research on guided wave (GW) simulations by Yang et al.^51^ suggests that the mesh size should be significantly smaller than one-sixth of the GW wavelength to satisfy the Courant–Friedrichs–Lewy (CFL) convergence conditions as described in the further subsection. Consequently, the maximum mesh size adopted for the HCM is limited to 0.5 mm based on the propagating group velocity. Additionally, a finer mesh is employed around sharp crack edges and along the abrasion depth to tackle the stress concentration and significant stress variation, as shown in Fig. 5.

To address the guided wave propagation problem involving multiphysics simulation, a Time-Dependent study is conducted to simulate the excitation, propagation, and response acquisition of guided waves (GW). The study uses a generalised HHT-α method with a time step (Δt) of 0.05 μs and a MUMPS (MUltifrontal Massively Parallel sparse direct Solver) solver with a memory allocation factor of 1.1 for the time-dependent analysis.

### Courant–Friedrichs–Lewy condition

The Courant–Friedrichs–Lewy (CFL) condition^52^ serves as a criterion for assessing the convergence of solutions in time-dependent studies. This criterion plays a pivotal role in determining the maximum permissible mesh size for the model and the appropriate time step to be utilised in time-dependent simulations. To ensure the CFL conditions are satisfied, the following steps are undertaken:

- *Group Velocity Comparison and Wave Mode Identification* The wave mode propagating in the specimen at the excited central frequency through an experimental analysis is first identified by comparing the group velocity of the first wave packet with the velocity in the dispersion curve derived from the SAFE formulation. To calculate group velocity, the time of arrival of the first wave packet at the receiver PZT with respect to the exciting Hanning pulse is estimated. Further, dividing the distance between the actuator and the receiver by the estimated time of arrival allows for the calculation of the group velocity of the wave mode.
- *Mesh Size Determination* Based on the group velocity, the maximum permissible mesh size is determined using the CFL criterion, as described in Eq. 1. This step ensures that the time step and spatial discretisation are consistent with the wave propagation characteristics.
- *Time Step Calculation* Once the mesh size was established, the maximum time step required to meet the CFL criterion was calculated and used in the numerical analysis to ensure accurate wave propagation.

#### Mesh and time step criteria

The CFL condition is as follows:

1 $$\Delta s \le \frac{{C_{g} }}{6f}\;{\text{and}}\;C = \frac{{C_{g} \Delta t}}{\Delta s} \le 1$$

where the dimensionless number *C* is called the Courant number and is considered 1 for the current study. *C*_*g*_ is the magnitude of the group velocity of the considered wave mode. Δt is the time step, and Δs is the mesh size.

### Material properties

The material properties of woven carbon fibres considered for the composite laminated hollow section, referred from the work of Ahmad et al.^53^, are presented in Table 1. As the considered material is woven carbon fibres, the fibres in each lamina are aligned in both in-plane directions (i.e., longitudinal and transverse) and possess equal elastic properties (E_11_ = E_22_), thereby considering the homogenised material model of fibres and epoxy. Further, the simulated PZTs are assigned the material properties described in Table 2, referred from the work of Qiu et al.^54^.

**Table 1:** Material property of carbon fibre reinforced woven composite.

E_11_ (GPa)	E_22_ (GPa)	E_33_ (GPa)	G_12_ (GPa)	G_13_ (GPa)	G_23_ (GPa)	ν_12_	ν_13_ = ν_23_	ρ (kg/m^3^)
51.4	51.4	11.7	4.42	3.90	3.90	0.09	0.10	1630

**Table 2:** Material property of piezoelectric sensor (PZT-5A).

Parameter	Values	
Relative permittivity	ε_11_	1730
	ε_33_	1700
Piezoelectric constant (× 10^–10^ C/N)	d_31_ = d_32_	− 1.71
	d_33_	3.74
	d_15_	5.84
Compliance coefficient (× 10^–12^ m^2^/N)	sE_11_	16.4
	sE_12_	− 5.74
	sE_13_	− 7.22
	sE_33_	18.8
	sE_55_	4.75
	sE_66_	4.43
Density (kg/m^3^)	ρ	7750
Elastic properties (GPa)	E_11_ = E_22_	60.97
	E_33_	53.19
Poison ratio	ν_13_ = ν_23_	0.4402
	ν_12_	0.35

## SAFE model for the analysis of guided wave dispersion in the HCM

**Fig. 6:**
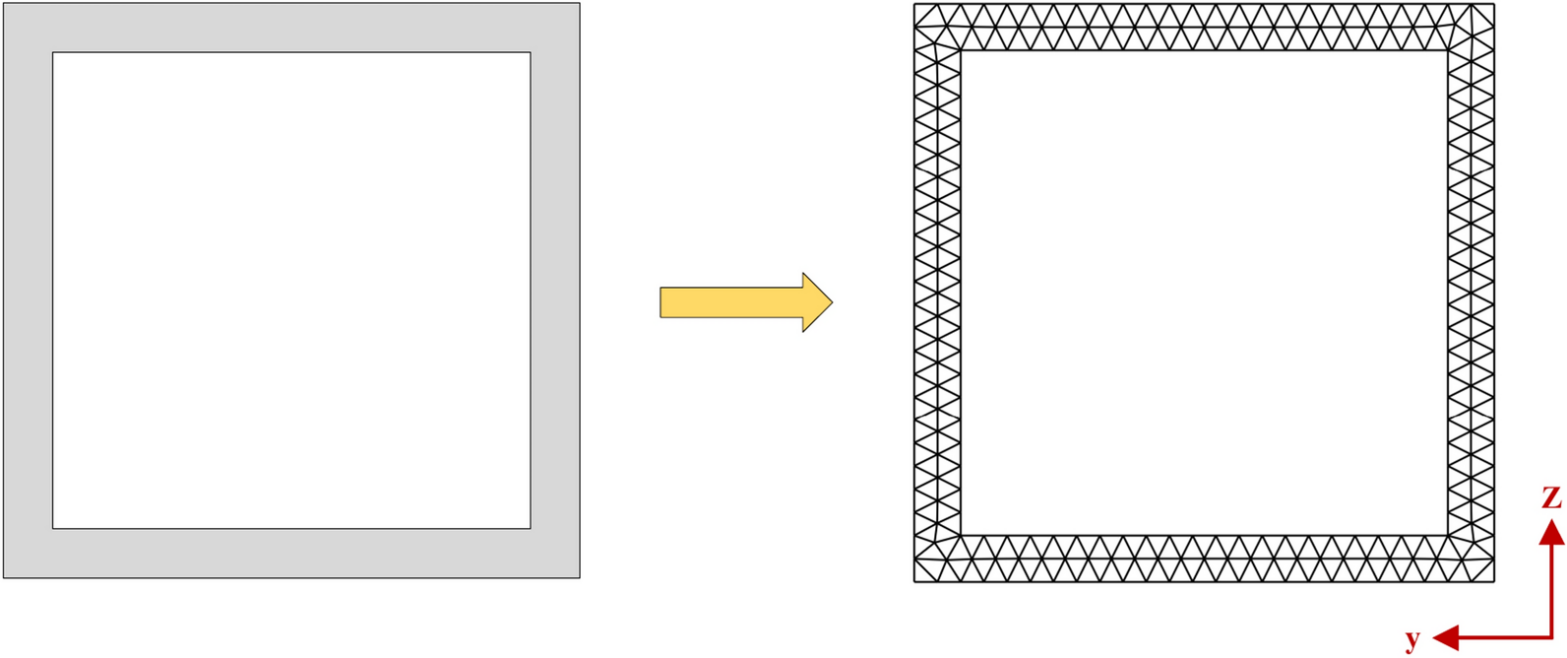
Cross section and finite element discretisation of Hollow Section.

A Semi-Analytical Finite Element (SAFE) formulation^55^ is developed for comprehending the dispersion characteristics of guided waves in the HCM assuming plain strain condition. The cross-section of a hollow composite member is discretised using a 3-noded triangular element, as shown in Fig. 6, and waves propagate in the *x*-direction, described by the exponential function *e*^*ikx*^ where *k* is the wavenumber of the Lamb wave. The dispersion curve is plotted by extracting the wavenumber *k* in the eigensystem using the eigs command in MATLAB 2018^®^.

The displacement (u^(e)^), strain (ɛ) and stress (σ) component at individual nodes of a triangular element by considering the exponential variation $e \, ^{i\omega t}$ is given by:

2 $$u^{{({\text{e}})}} = \left\{ {u_{{\text{x}}}^{{({\text{e}})}} \, u_{{\text{y}}}^{{({\text{e}})}} \, u_{{\text{z}}}^{{({\text{e}})}} } \right\}^{{\text{T}}} {\text{e}}^{{{\text{i}}({\text{kx}} - \omega {\text{t}})}} ;\;\varepsilon = \left\{ {\varepsilon_{{{\text{xx}}}} \, \varepsilon_{{{\text{yy}}}} \, \varepsilon_{{{\text{zz}}}} \, \varepsilon_{{{\text{yz}}}} \, \varepsilon_{{{\text{xz}}}} \, \varepsilon_{{{\text{xy}}}} } \right\}^{{\text{T}}} ;\;\sigma = \left\{ {\sigma_{{{\text{xx}}}} \, \sigma_{{{\text{yy}}}} \, \sigma_{{{\text{zz}}}} \, \sigma_{{{\text{yz}}}} \, \sigma_{{{\text{xz}}}} \, \sigma_{{{\text{xy}}}} } \right\}^{{\text{T}}}$$

The linear strain is given by

3 $$\varepsilon_{L}^{(e)} { = }\left\{ {\begin{array}{*{20}c} {\frac{{\partial u_{x} }}{\partial x}} \\ {\frac{{\partial u_{y} }}{\partial y}} \\ {\frac{{\partial u_{z} }}{\partial z}} \\ {\frac{{\partial u_{y} }}{\partial z} + \frac{{\partial u_{z} }}{\partial y}} \\ {\frac{{\partial u_{x} }}{\partial z} + \frac{{\partial u_{z} }}{\partial x}} \\ {\frac{{\partial u_{x} }}{\partial y} + \frac{{\partial u_{y} }}{\partial x}} \\ \end{array} } \right\} \, = \, \left( {\left[ {\begin{array}{*{20}c} {\frac{\partial }{\partial x}} & 0 & 0 \\ 0 & 0 & 0 \\ 0 & 0 & 0 \\ 0 & 0 & 0 \\ 0 & 0 & {\frac{\partial }{\partial x}} \\ 0 & {\frac{\partial }{\partial x}} & 0 \\ \end{array} } \right] + \left[ {\begin{array}{*{20}c} 0 & 0 & 0 \\ 0 & {\frac{\partial }{\partial y}} & 0 \\ 0 & 0 & 0 \\ 0 & 0 & {\frac{\partial }{\partial y}} \\ 0 & 0 & 0 \\ {\frac{\partial }{\partial y}} & 0 & 0 \\ \end{array} } \right] + \left[ {\begin{array}{*{20}c} 0 & 0 & 0 \\ 0 & 0 & 0 \\ 0 & 0 & {\frac{\partial }{\partial z}} \\ 0 & {\frac{\partial }{\partial z}} & 0 \\ {\frac{\partial }{\partial z}} & 0 & 0 \\ 0 & 0 & 0 \\ \end{array} } \right]} \right)\left\{ {\begin{array}{*{20}c} {u_{{\text{x}}}^{(e)} } \\ {u_{y}^{(e)} } \\ {u_{z}^{(e)} } \\ \end{array} } \right\}$$

where

4 $${\upvarepsilon }_{{\text{L}}}^{{\text{(e)}}} { = }\left[ {{\text{L}}_{{\text{x}}} \frac{\partial }{\partial x} + {\text{L}}_{{\text{y}}} \frac{\partial }{\partial y} + {\text{L}}_{{\text{z}}} \frac{\partial }{\partial z}} \right]{\text{u}}^{{\text{(e)}}}$$

Substituting the displacement field in Eq. (4) reduces to

5 $$\varepsilon_{L}^{(e)} = {\text{ (B}}_{1} {\text{ + ikB}}_{2} ){\text{q}}^{(e)} e^{i(kx - \omega t)}$$

where

6 $${\text{B}}_{{1}} {\text{ = L}}_{{\text{y}}} {\text{N}}_{{\text{,y}}} + {\text{L}}_{{\text{z}}} {\text{N}}_{{\text{,z}}} \;{\text{and}}\;{\text{B}}_{{2}} {\text{ = L}}_{{\text{x}}} {\text{N}}$$

where N is the linear displacement interpolation function matrix.

Employing the virtual work principle i.e., $\delta H = \, \int_{{t_{1} }}^{{t_{2} }} {\left[ {\int_{V} {\delta \left( {\varepsilon_{L}^{T} } \right)} \, C \, \varepsilon_{L} \, dV + \int_{V} {\delta \left( {u^{T} } \right) \, } \rho_{e} \mathop u\limits^{..} dV \, } \right]} \, dt = \, 0$, the following governing equation of wave is obtained,

7 $$\left[ {{\text{K}}_{1} + {\text{kK}}_{2} + {\text{k}}^{2} {\text{K}}_{3} - \omega^{2} {\text{M}}} \right]{\text{Q}} = \, 0$$

8 $${\text{K}}_{1} = {\text{T}}^{{\text{T}}} \left( {\bigcup\limits_{e = 1}^{{{\text{n}}_{{{\text{el}}}} }} {\int\limits_{ - 1}^{1} {{\text{B}}_{1}^{{\text{T}}} {\text{C}}_{{({\text{e}})}} {\text{B}}_{1} {\text{d}}\xi } } } \right){\text{T}};\; - {\text{iK}}_{2} = {\text{T}}^{{\text{T}}} \left( {\bigcup\limits_{e = 1}^{{{\text{n}}_{{{\text{el}}}} }} {\int\limits_{ - 1}^{1} {\left( {{\text{B}}_{1}^{{\text{T}}} {\text{C}}_{{({\text{e}})}} {\text{B}}_{2} - {\text{B}}_{2}^{{\text{T}}} {\text{C}}_{{({\text{e}})}} {\text{B}}_{1} } \right){\text{d}}\xi } } } \right){\text{T}}$$

9 $${\text{K}}_{3} = {\text{T}}^{{\text{T}}} \left( {\bigcup\limits_{{{\text{e}} = 1}}^{{{\text{n}}_{{{\text{el}}}} }} {\int\limits_{ - 1}^{1} {{\text{B}}_{2}^{{\text{T}}} {\text{C}}_{{({\text{e}})}} {\text{B}}_{2} {\text{d}}\xi } } } \right){\text{T}};\;{\text{M}} = {\text{T}}^{{\text{T}}} \left( {\bigcup\limits_{{{\text{e}} = 1}}^{{{\text{n}}_{{{\text{el}}}} }} {\int\limits_{ - 1}^{1} {{\text{N}}^{{\text{T}}} \rho_{{({\text{e}})}} {\text{Nd}}\xi } } } \right){\text{T}}$$

Here, C is the constitutive matrix of the laminate and T is the Hermitian matrix such that T.T^T^ = I.

Two variables, the wavenumber *k* and circular frequency *ω*, are present in Eq. (7). Therefore, this equation is solved by fixing the frequency *ω* and estimating the corresponding value for the wavenumber *k*. Hence, Eq. (7) is rewritten to a linear form as

10 $$\left[ {{\text{A}} - {\text{k}} \cdot {\text{B}}} \right]\left[ {\begin{array}{*{20}c} {\text{Q}} \\ {{\text{kQ}}} \\ \end{array} } \right]{ = 0}$$

where

11 $${\text{A = }}\left[ {\begin{array}{*{20}c} {0} & {{\text{K}}_{{1}} - {\upomega }^{{2}} {\text{M}}} \\ {{\text{K}}_{{1}} - {\upomega }^{{2}} {\text{M}}} & {{\text{K}}_{{2}} } \\ \end{array} } \right]\;{\text{B = }}\left[ {\begin{array}{*{20}c} {{\text{K}}_{{1}} - {\upomega }^{{2}} {\text{M}}} & {0} \\ {0} & { - {\text{K}}_{{3}} } \\ \end{array} } \right]$$

Equation (10) is the eigenvalue problem with wavenumber ‘*k*’ as the eigen value. The phase velocity of the *m*^*th*^ mode for the corresponding frequency *ω* is given by *C*_*pm*_ = *ω/k*_*m*_*.*

Group velocity dispersion relation is a basically a rate of change of frequency (*ω*) with respect to wavenumber (*k*). Therefore, by differentiating Eq. (7), and rearranging the terms to represent the group velocity equation, results in

12 $${\text{C}}_{{\text{g}}} = \frac{\partial \omega }{{\partial {\text{k}}}} = \frac{{{\text{Q}}_{{\text{L}}}^{{\text{T}}} \left( {{\text{K}}_{2} + 2{\text{kK}}_{3} } \right){\text{Q}}_{{\text{R}}} }}{{2\omega {\text{Q}}_{{\text{L}}}^{{\text{T}}} {\text{MQ}}_{{\text{R}}} }}$$

From Eq. (12), it is clear that the group velocity of the propagating wave is the function of (k − ω) pairs. Q_R_ and Q_L_ are the right and left eigenvectors, respectively.

For the generalised structures, the orthogonality relationships of the eigenvectors are applied to extract the Lamb wave modes^56^. Therefore, the B-orthogonality principle of eigenvectors is given by

13 $${\text{Q}}_{{\text{R}}}^{{\text{T}}} \left( \omega \right)\left[ {\text{B}} \right]{\text{Q}}_{{\text{R}}} \left( \omega \right) \ne 0$$

## Experimental setup and details

**Fig. 7:**
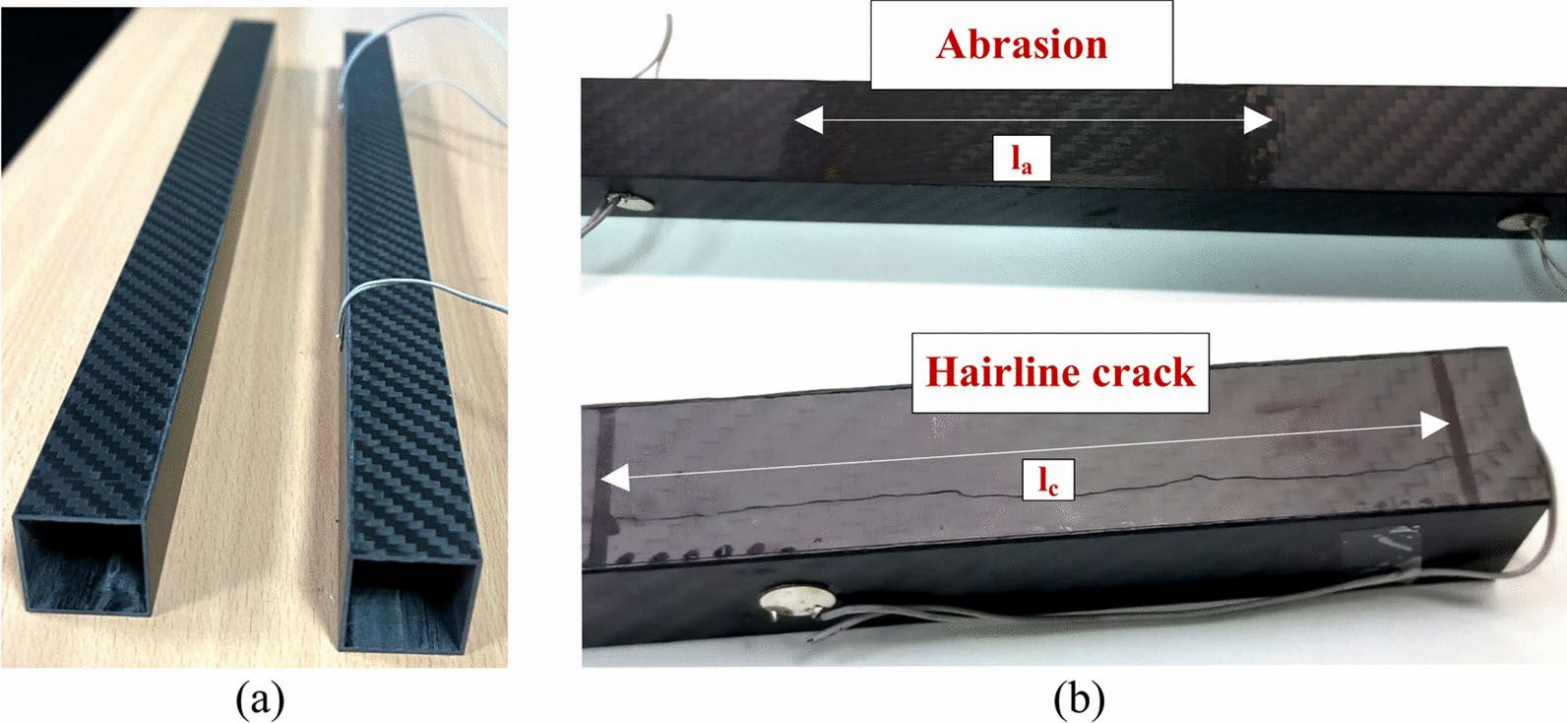
(**a**) Hollow composite square section (**b**) abrasion damage and Hairline crack considered in the experimental studies.

**Fig. 8:**
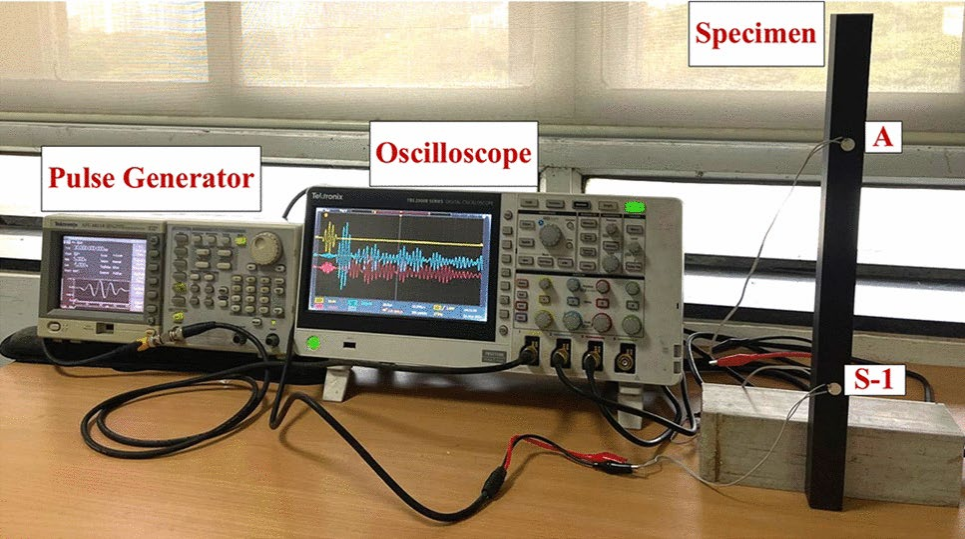
Experimental setup for guided wave propagation investigation in HCM.

This section details the experimental setup used to investigate wave propagation in the square HCM in order to validate the simulation results. The experiment is conducted on square HCM fabricated using woven carbon fibres, as depicted in Fig. 7a, with varying widths of abrasion and hairline crack shown in Fig. 7b. Wave propagation investigations are carried out using three circular PZT wafers (A, S1 and S2), a pulse generator, and an oscilloscope. The subsequent subsections briefly describe the setup details, as illustrated in Fig. 8.

The HCM considered in the experiment has a dimension of 25 × 25 × 410 mm^3^, with a section thickness of 1 mm, as shown in Fig. 7a. Further, two types of damage such as abrasion and hairline crack, are considered. In the experiment, the uniform rectangular abrasion is introduced by filing the surface of interest with 60-grit sandpaper, followed by smoothing with 80-grit sandpaper to ensure a uniform abraded surface. The hairline crack, on the other hand, is a pre-existing manufacturing defect present in one of the procured samples, aligned parallel to the surface of the attached PZTs. In this experiment, the guided wave study is conducted using three circular piezoelectric patches (PZTs) of 10 mm diameter and thickness of 0.4 mm, in which two PZTs (S1 and S2) act as receivers and PZT ‘A’ as an actuator. The actuator PZT ‘A’ is excited with a 5-cycle sine-modulated Hanning window with a central frequency of 150 kHz and 10 Vpp (Volt peak-to-peak) using a Tektronix AFG1022 pulse generator, and the responses are captured through PZTs using a Tektronix TBS 2000B series digital oscilloscope. The Hanning window generated is passed to the actuators and the oscilloscope through T-connectors. The response is received through PZT ‘S1’ and ‘S2’ mounted at a distance of 200 mm from the actuator through an oscilloscope. Subsequently, the response signal is acquired through a receiver transducer and recorded using an oscilloscope’s averaging mode (256 times), employing a sampling frequency of 10 MHz. Further, the obtained signal is denoised by the mean of wavelet coefficients soft thresholding method with a single global positive threshold. Orthogonal Daubechies wavelet (db5) with the 5th level of decomposition is employed for denoising. The denoised signal is then analysed to inspect the variations in wave features to compare the response obtained from the numerical simulation with the experimental outcomes to validate the numerical model as discussed in “Comparison studies and dispersion analysis” section. The same specimen dimensions and PZT configurations are used for further parametric investigation, as detailed in “Conclusions” section.

## Signal features and parametric indices

This section covers the signal processing techniques applied to analyse the changes in the scattered guided waveform features resulting from the change in different parameters obtained from various PZT receivers. Damage indices (DIs) are estimated to assess how the variation correlates with changes in different damage parameters. In this study, features are elicited either directly from the waveform or through the statistical approach. The signals are compared with the amplitude, energy content and wavelet coefficients across the frequency range. Various DIs are utilised to quantify the damage state in the HCM using these waveform parameters. The DIs based on energy and wavelet coefficients are newly proposed for the current investigation, while the SSD and NCM DIs are referenced appropriately from the existing literature. Moreover, the “normalised correlation moment (NCM)” damage index is used for statistical comparison between the signal in the current state and the signal in the reference state. Finally, to distinguish between abrasion and hairline crack signals, the scattered signals are compared statistically using the variance and mean of the continuous wavelet transform (CWT) peak within an appropriate frequency range. The details of the various DI employed are given below:

### Damage index based on energy

The baseline-dependent damage index, based on the energy content of the received signal in both the damaged and pristine states of the hollow section, is defined as follows:

14 $${\text{DI}}_{{\text{E}}} = 1 \, - \, \frac{{\sum\nolimits_{t = 0}^{T} {S_{d} {(}t{)}^{2} } }}{{\sum\nolimits_{t = 0}^{T} {S_{p} {(}t{)}^{2} } }}$$

where *S*_*d*_ and *S*_*p*_ are the amplitude of the received waveform in the damaged and pristine state of the hollow section. This DI quantifies the energy content of the received signal in both damaged and pristine states.

### Damage index based on wavelet coefficient

The damage index based on the CWT coefficient of the received waveform over a frequency domain from *f*_*1*_ to *f*_*2*_ is considered and estimated for both the damaged and pristine states of the hollow section. Morlet wavelet is employed for waveform analysis. The signal is analysed by calculating the baseline-dependent time–frequency domain index within the specified frequency range over the total acquisition time (*T*), as detailed below:

15 $${\text{DI}}_{{\text{w}}} = 1 \, - \, \frac{{\left. {\sum\nolimits_{t = 0}^{T} {C_{d} {(}t{)}} } \right|_{{f_{1} }}^{{f_{2} }} }}{{\left. {\sum\nolimits_{t = 0}^{T} {C_{p} {(}t{)}} } \right|_{{f_{1} }}^{{f_{2} }} }}$$

In the current study, the CWT coefficient is considered over a frequency range of *f*_*1*_ = 90 kHz to *f*_*2*_ = 210 kHz, as it corresponds to the frequency range of the input 5-cycle sine-modulated Hanning window with 150 kHz central frequency. This approach is particularly advantageous because it allows for the examination of localised frequency changes over time, which can be indicative of specific types of damage. Further, it is beneficial for capturing transient behaviour and frequency shifts that may not be apparent in time-domain analyses alone.

### Damage index based on signal sum of square differences

Another index based on the signal sum of square differences (SSD) referred from the work of Loendersloot et al.^57^ is considered in the current investigation, which is based on the difference of the waveform response obtained through PZTs in the pristine and damage states, which is defined as follows:

16 $${\text{DI}}_{{{\text{SSD}}}} = \frac{1}{{1 + \frac{{\sum\nolimits_{t = 0}^{T} {\left( {S_{p} \left( t \right) - S_{d} \left( t \right)} \right)^{2} } }}{{\sum\nolimits_{t = 0}^{T} {S_{p}^{2} \left( t \right)} }}}} \,$$

where *S*_*d*_ and *S*_*p*_ are the amplitude of the received waveform in the damaged and pristine state of the hollow section. This DI provides a straightforward quantitative measure of damage by highlighting deviations in signal amplitude. The SSD approach is particularly effective for detecting subtle changes that may occur due to minor damage, making it a useful tool for early detection.

### Normalised correlation moment (NCM)

A baseline-dependent time-domain parametric index, referred to as normalised correlation moment (NCM), developed by Torkamani et al.^58^, is employed based on the cross-correlation of the baseline and observation waveforms acquired from the structure. Therefore, the time-domain parametric index ‘normalised correlation moment (NCM)’ is defined as follows:

17 $$NCM = \, \frac{{\int_{\tau = 0}^{{\tau {\text{ = T}}_{{\text{a}}} }} {\tau^{n} \left| {r_{xy} \left( \tau \right)} \right|d\tau - \int_{\tau = 0}^{{\tau {\text{ = T}}_{{\text{a}}} }} {\tau^{n} \left| {r_{xx} \left( \tau \right)} \right|d\tau } } }}{{\int_{\tau = 0}^{{\tau {\text{ = T}}_{{\text{a}}} }} {\tau^{n} \left| {r_{xx} \left( \tau \right)} \right|d\tau } }}$$

where *r*_*xy*_ is the cross-correlation of the baseline signal and comparison signal, *r*_*xx*_ is the autocorrelation, *T*_*a*_ is the duration of response acquisition, *τ* is referred to as the ‘lag’ parameter, and *n* is the order of the statistical moment, which is taken as 0.01. This index is significant as it quantifies how closely related the current state is to an undamaged reference state, thus providing a statistical measure of damage severity.

### Variance of the CWT peak

A baseline-free time–frequency domain index, the variance of the peak of CWT coefficients within a specified frequency range over a total acquisition time (*T*), is used. In this study, the variance is analysed within the frequency range of *f*_*1*_ = 90 kHz to *f*_*2*_ = 210 kHz. Thus, the time–frequency domain parametric index known as ‘Variance’ is expressed as follows:

18 $$Variance = \, \left. {\frac{{\sum\nolimits_{n = 0}^{N} {\left| {P_{n} - \overline{P} } \right|}^{2} }}{N}} \right|_{{f_{1} }}^{{f_{2} }}$$

where *P* is the individual CWT coefficient peak, $\overline{P }$ is the mean of the obtained CWT coefficient peak over a frequency range of *f*_*1*_ to* f*_*2,*_ and *N* is the total number of peaks obtained over a frequency range.

## Comparison studies and dispersion analysis

This section presents comparative studies aimed at verifying the accuracy and efficiency of the developed finite element model by analysing the scattered guided waveform in the HCM. Initially, a brief investigation is performed on the experimental response to assess how the excitation frequency affects the peak amplitude of the scattered response. After identifying the optimal excitation frequency, comparison studies are conducted on various damage types. The results received from the COMSOL Multiphysics® are compared with the experimental response for various lengths of surface abrasion and hairline crack obtained through both the mounted receiver PZTs. This study primarily focuses on the arrival time of the different wave packets, with less emphasis placed on the wave packet amplitude due to the influence of other factors. Furthermore, dispersion curves obtained through the Semi-Analytical Finite Element (SAFE) formulation is plotted to analyse the dispersive characteristics of hollow sections. A detailed discussion of the comparative and dispersion analyses are presented in the following subsections.

### Effect of excitation frequency on the peak amplitude of GW response

**Fig. 9:**
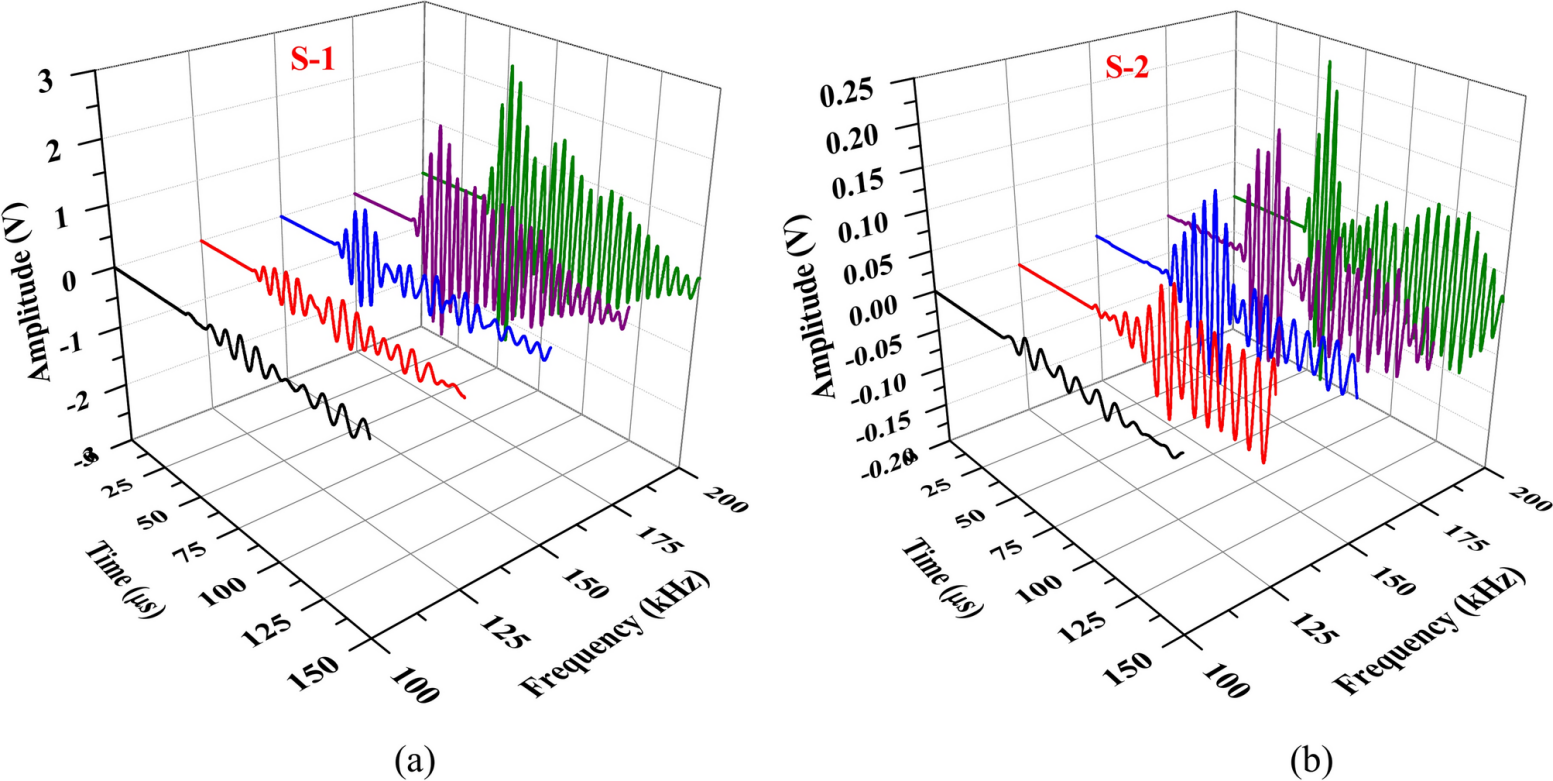
Time domain wave response acquired through PZT (**a**) S-1 and (**b**) S-2 in experiment for different excitation frequencies in the HCM.

**Fig. 10:**
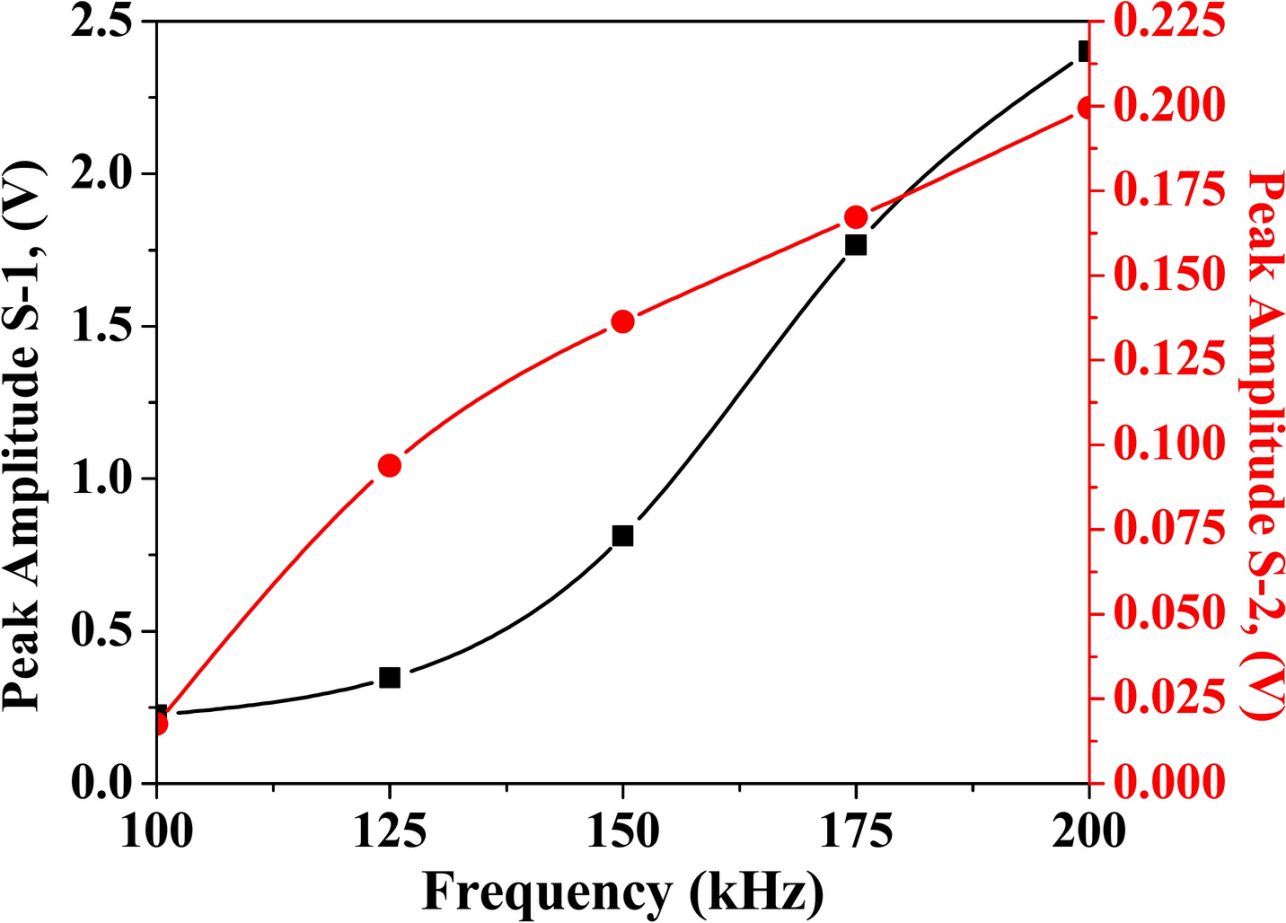
Frequency modulation plot of the HCM.

This section covers studies examining the effect of excitation frequency on the behaviour of various wave modes, which is a critical step before performing a detailed parametric wave propagation analysis. This segment of the study involves plotting the frequency-amplitude modulation curve to assess the impact of excitation frequency on the peak amplitude of the response wave. The current research focuses on a woven fabric hollow composite section. A 5-cycle Hanning window is excited through one actuator, and the corresponding scattered response is acquired through PZTs S-1 and S-2 from the experimental investigation. The excitation frequency range varies from 100 to 200 kHz at an interval of 25 kHz, with the peak amplitudes of the scattered response analysed for each frequency. Figure 9 illustrates the wave response acquired through S-1 and S-2 PZTs at different excitation frequencies, while Fig. 10 depicts the frequency modulation curve. Analysis of the modulation plot highlights that the peak amplitude of the waveform is enhanced with rising frequency. It is crucial to consider not only the peak response but also to differentiate different wave mode separations within the response. So, after a thorough examination, it is noticed that when the input wave is excited at a central frequency of 150 kHz, the scattered response exhibits clearly separated and identifiable wave packets. Consequently, for the subsequent investigations, the actuator PZT is excited with a sine-modulated Hanning pulse at 150 kHz.

### Comparison study- 1

**Fig. 11:**
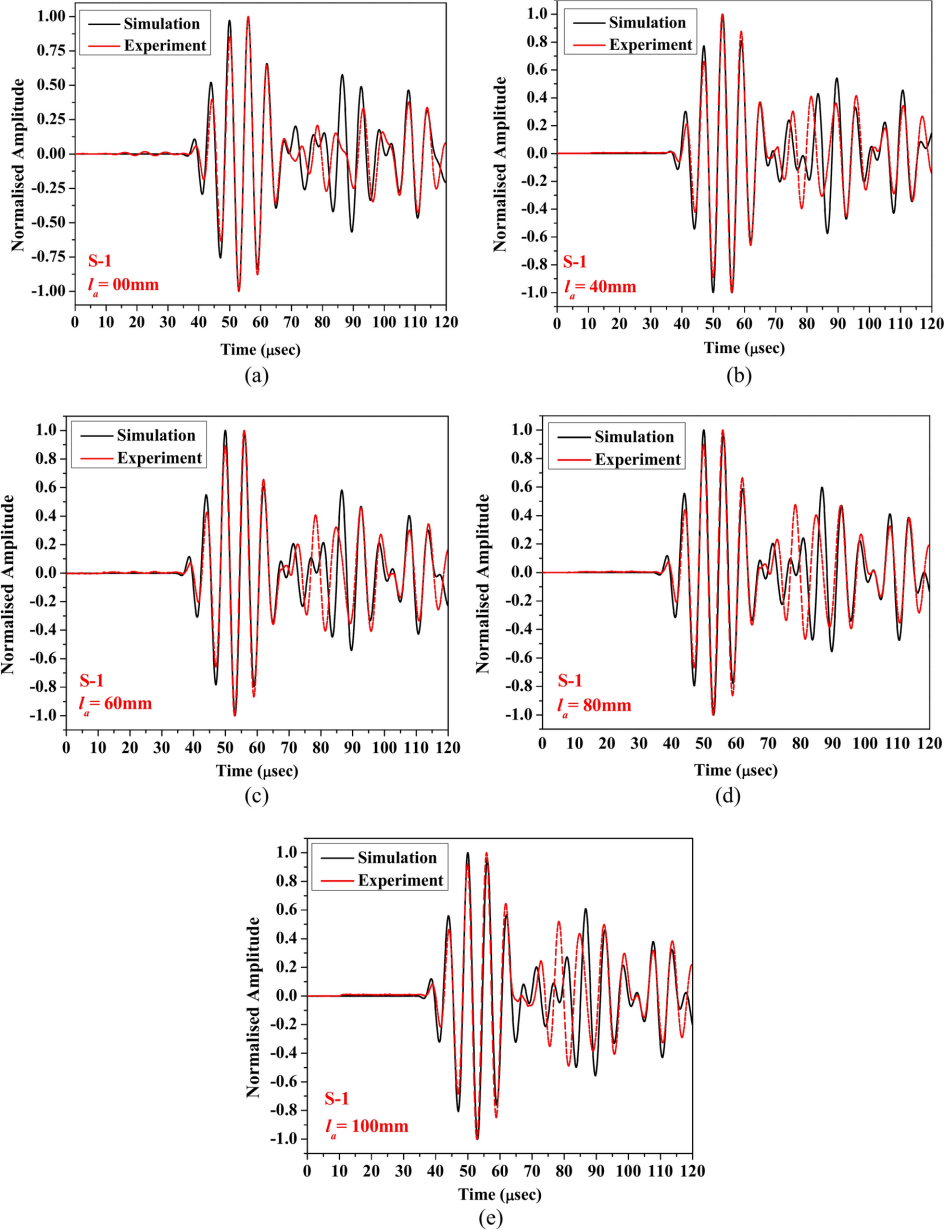
Waveform of the response acquired through S-1 for (**a**) healthy state, (**b**) abrasion length (*l*_*a*_) of 40 mm, (**c**) *l*_*a*_ = 60 mm, (**d**) *l*_*a*_ = 80 mm, and (**e**) *l*_*a*_ = 100 mm obtained experimentally and numerically.

**Fig. 12:**
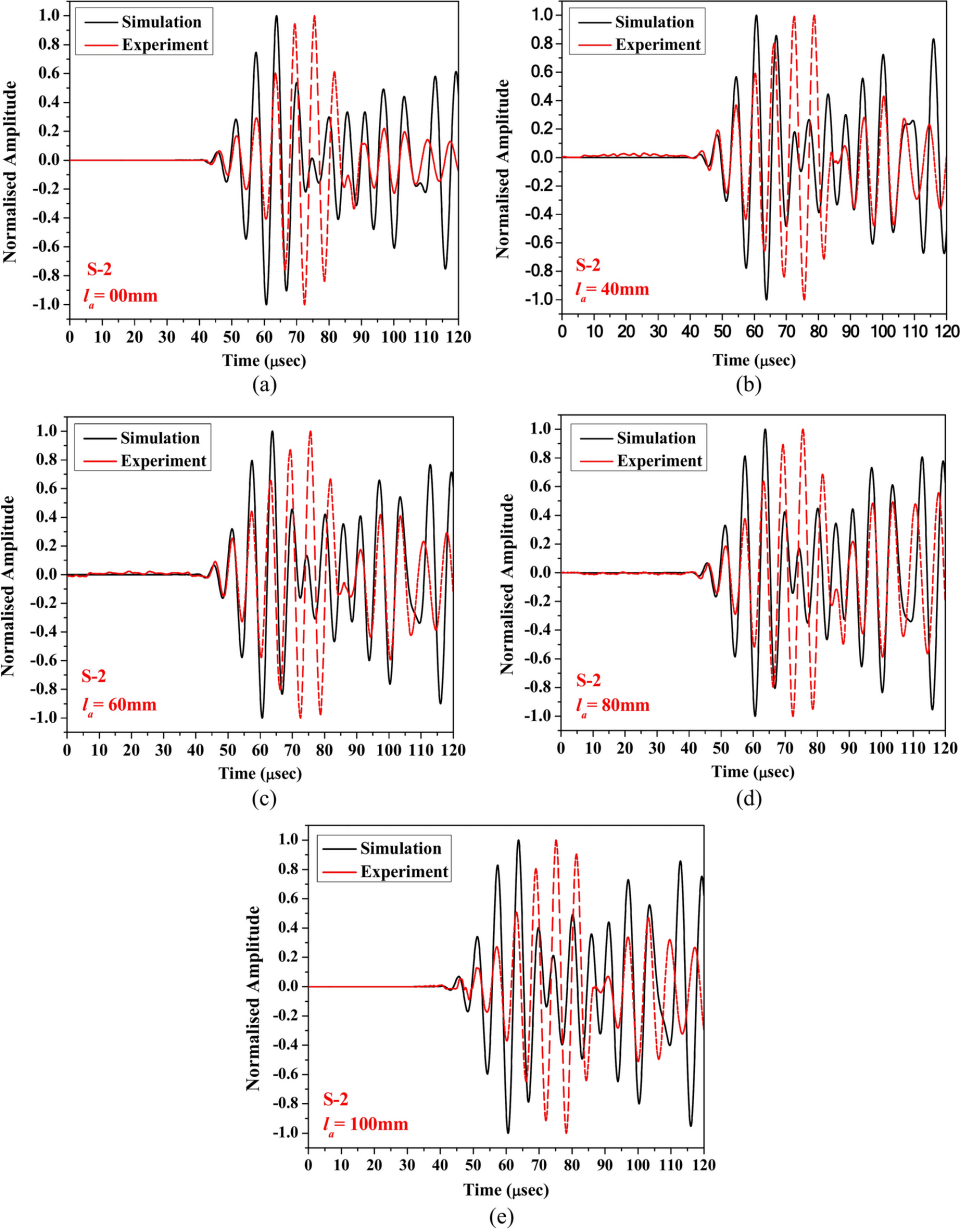
Waveform of the response acquired through S-2 for (**a**) healthy state, (**b**) abrasion length (*l*_*a*_) of 40 mm, (**c**) *l*_*a*_ = 60 mm, (**d**) *l*_*a*_ = 80 mm, and (**e**) *l*_*a*_ = 100 mm obtained experimentally and numerically.

To evaluate the accuracy of the numerical model developed using COMSOL Multiphysics®, this section compares the numerical results for different lengths of abrasion acquired through the surface-mounted PZTs with the experimental responses. The investigation focuses on scattered response in HCM through two receivers (S1 and S2) bonded on opposite faces of the section. To address the difference in amplitude between the simulated and experimental responses due to the omission of damping in the numerical model, the obtained data were normalised using Min–Max normalisation^59^. This normalisation allowed for a more equitable comparison of the responses across five distinct abrasion lengths (0 mm, 40 mm, 60 mm, 80 mm, and 100 mm) within a time-of-flight window of 120 μs while preserving the relative changes due to damage as depicted in Figs. 11 and 12. The outcome shows that irrespective of the abrasion lengths, the initial packet of the response acquired through receiver PZT S-1 obtained from numerical simulation are in good agreement with the experimental data. However, the wave packet in the time range of 65 μs to 85 μs shows a slight difference compared to the experimental response. Further, in the case of the response acquired through PZT S-2, the numerical response pattern closely matches the experimental response, irrespective of the abrasion length. Nevertheless, in the experimental waveform, an additional packet is observed to overlap with the first packet of the acquired waveform, which is absent in the numerical response. This additional packet likely arises from secondary wave reflections, mode conversions, or other complexities introduced by material heterogeneities, geometric factors, and PZT coupling mechanisms in addition to the surface-to-surface bond anomalies during manufacturing. These factors are quite complex to infer and fully replicate in the FE model and contribute to the observed differences. Despite this extra packet, the numerical and experimental responses are largely consistent, regardless of the HCM’s damage state. The FE model has been validated against ideal configurations to ensure its accuracy in simulating wave propagation and damage-wave interaction mechanisms.

**Fig. 13:**
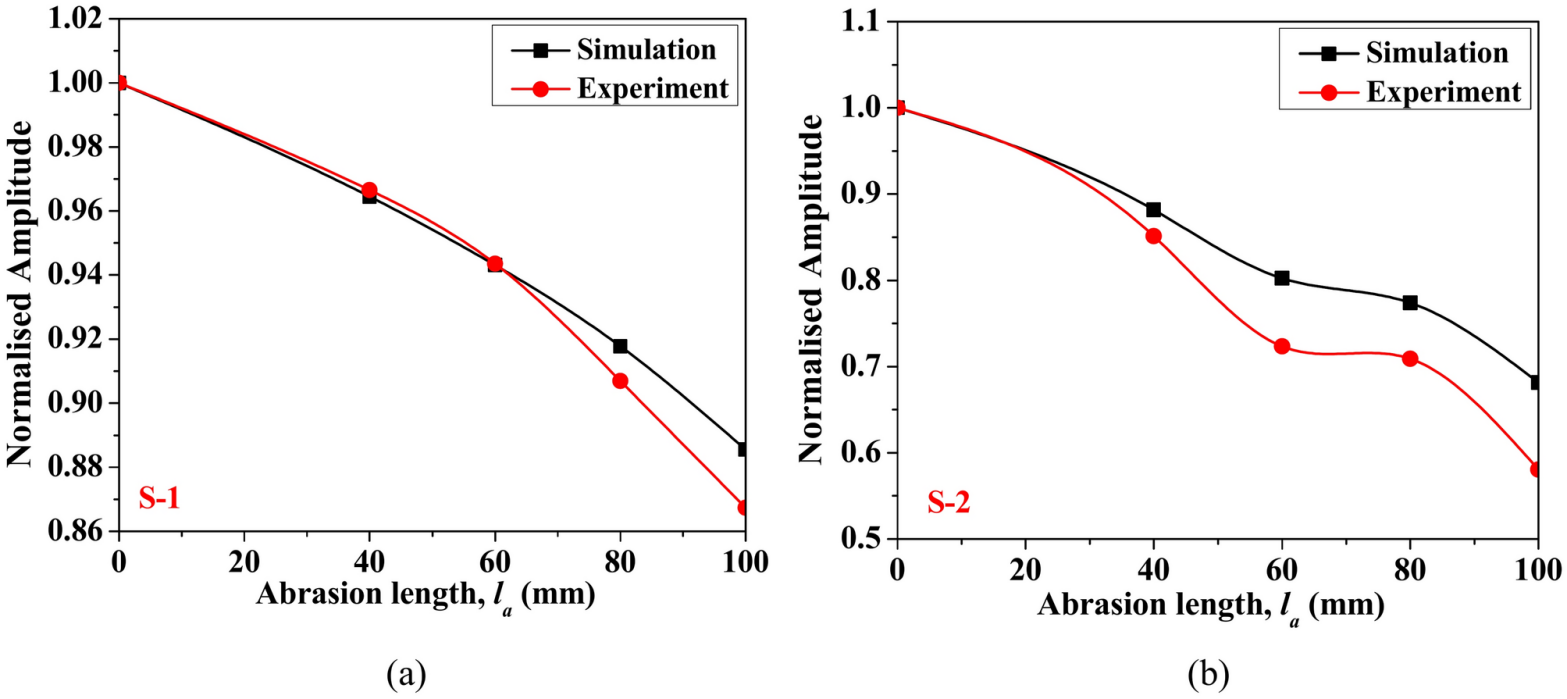
Variation of the normalised peak amplitude of the response obtained for different abrasion lengths acquired through (**a**) PZT S-1 and (**b**) PZT S-2.

Further, the variation in the peak amplitude of the first wave packet response, measured using PZT sensors S-1 and S-2 for different abrasion lengths, are compared in its normalised form by dividing the peak amplitude of the first wavepacket of the response for various abrasion lengths with the peak amplitude of the response for the healthy state, as shown in Fig. 13. The plot indicates that the amplitude of the response obtained through both experiment and simulation reduces with the increase in the abrasion size. However, the reduction in peak amplitude at larger abrasion sizes is marginally more in the experimental response compared to the simulated one. This difference in the rate of amplitude reduction can be attributed to the exclusion of damping parameters in the numerical model.

**Fig. 14:**
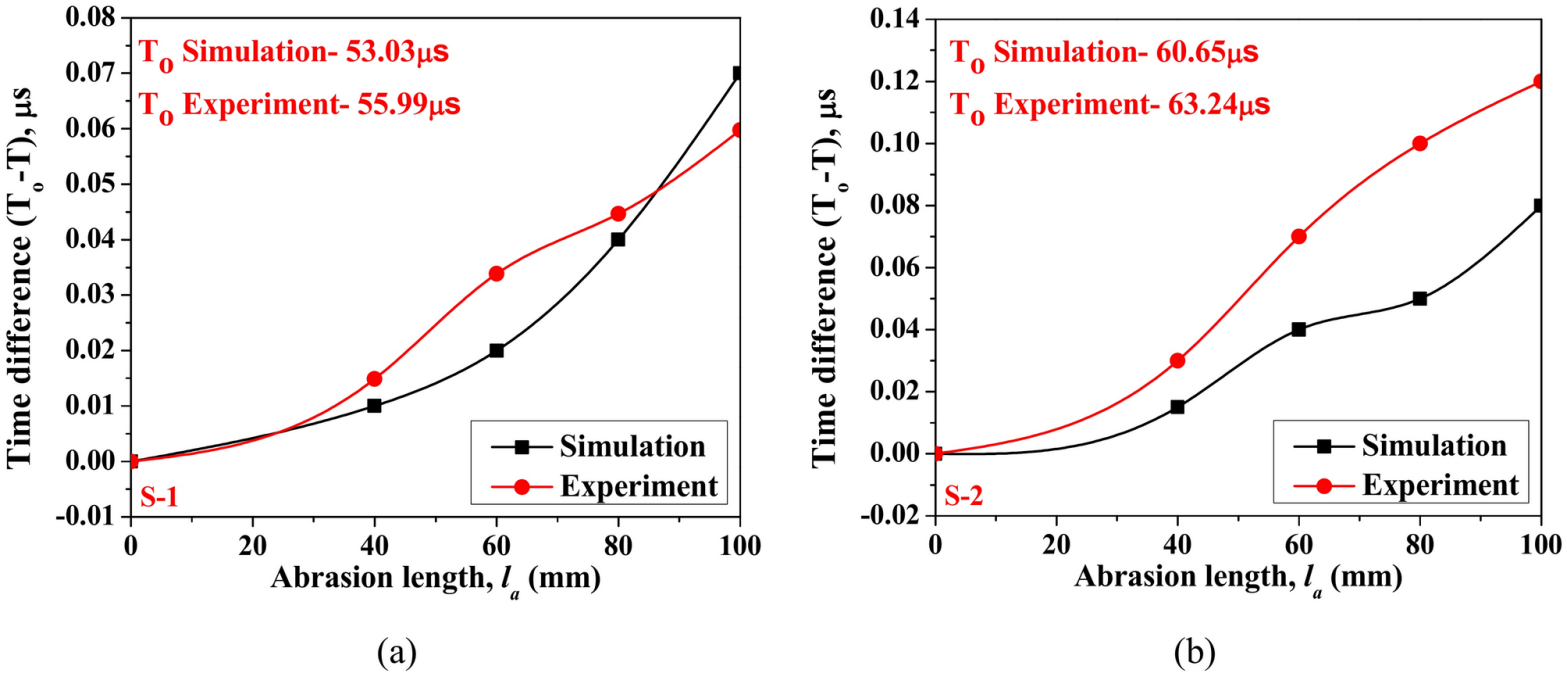
Advance in the time of arrival of the peak amplitude of response with respect to healthly state obtained for different abrasion lengths acquired through (**a**) PZT S-1 and (**b**) PZT S-2.

The advances in time of arrival (ToA) of the peak amplitude of response with respect to the healthy state signal are plotted in Fig. 14 for different abrasion lengths acquired through both PZTs. The study shows that the peak of the first wave packet in the response advances by a minimal amount with the increase in the abrasion size irrespective of the acquired PZT response, highlighting a slight rise in the group velocity. However, the ToA of the peak of the first wavepacket of response obtained through simulation advances by a small amount when compared to the experimental response. This consistency validates the use of the current FE model for conducting further detailed parametric investigations.

### Comparison study-2

**Fig. 15:**
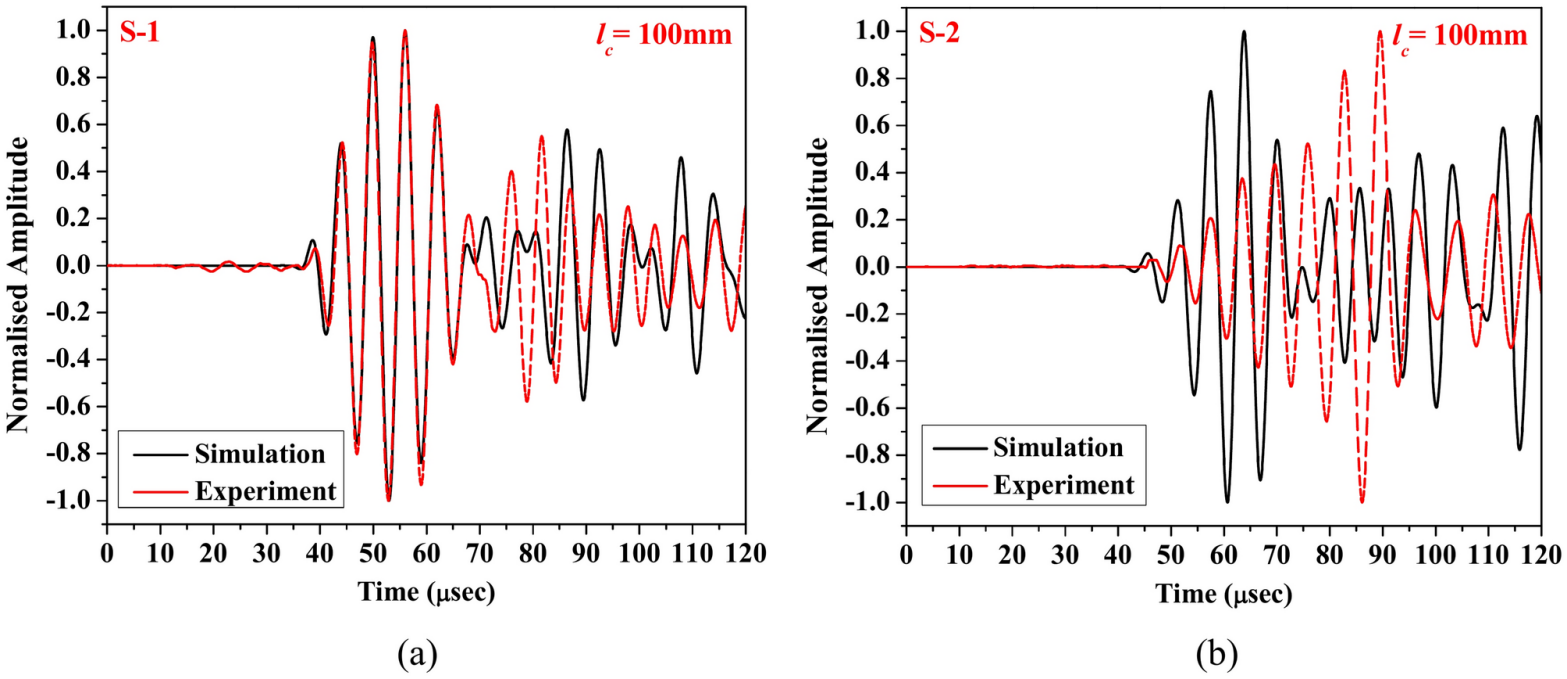
Waveform of the response acquired through (**a**) S-1 and (**b**) S-2, obtained experimentally and numerically for HCM with a hairline crack of 100 mm.

In addition to validating the signal acquired through experimental and numerical studies for various abrasion lengths in the preceding section. This section includes an analysis to investigate the closeness of the numerical response obtained through numerical study with the experimental response for a hairline crack of 100 mm length, as shown in Fig. 15. From the figure, it is noticed that the wavepacket of the response obtained through both the PZTs extracted through numerical simulation has a similar time of flight to that of the experimental response. However, a slight difference in the wave amplitude is noticed at the higher time duration. Additionally, the experimental response acquired through PZT S-2 shows an additional wavelet combined with the first mode wavepacket, which is not noticed in the response obtained through simulation. Despite this additional wavepacket, the numerical waveform obtained in the HCM with hairline crack agrees with the experimental outcome and hence develops the confidence to conduct a detailed parametric investigation.

### Guided wave dispersion characteristics

This part presents the dispersion curves generated through the Semi-Analytical Finite Element (SAFE) formulation^55^ described in “SAFE model for the analysis of guided wave dispersion in the HCM” section. This analysis investigates the variations in the phase velocity and group velocity of the various wave modes in a 1 mm thin composite hollow square section. The dispersion curve is plotted along the steering angle of 0° by varying the excitation frequency upto 300 kHz.

**Fig. 16:**
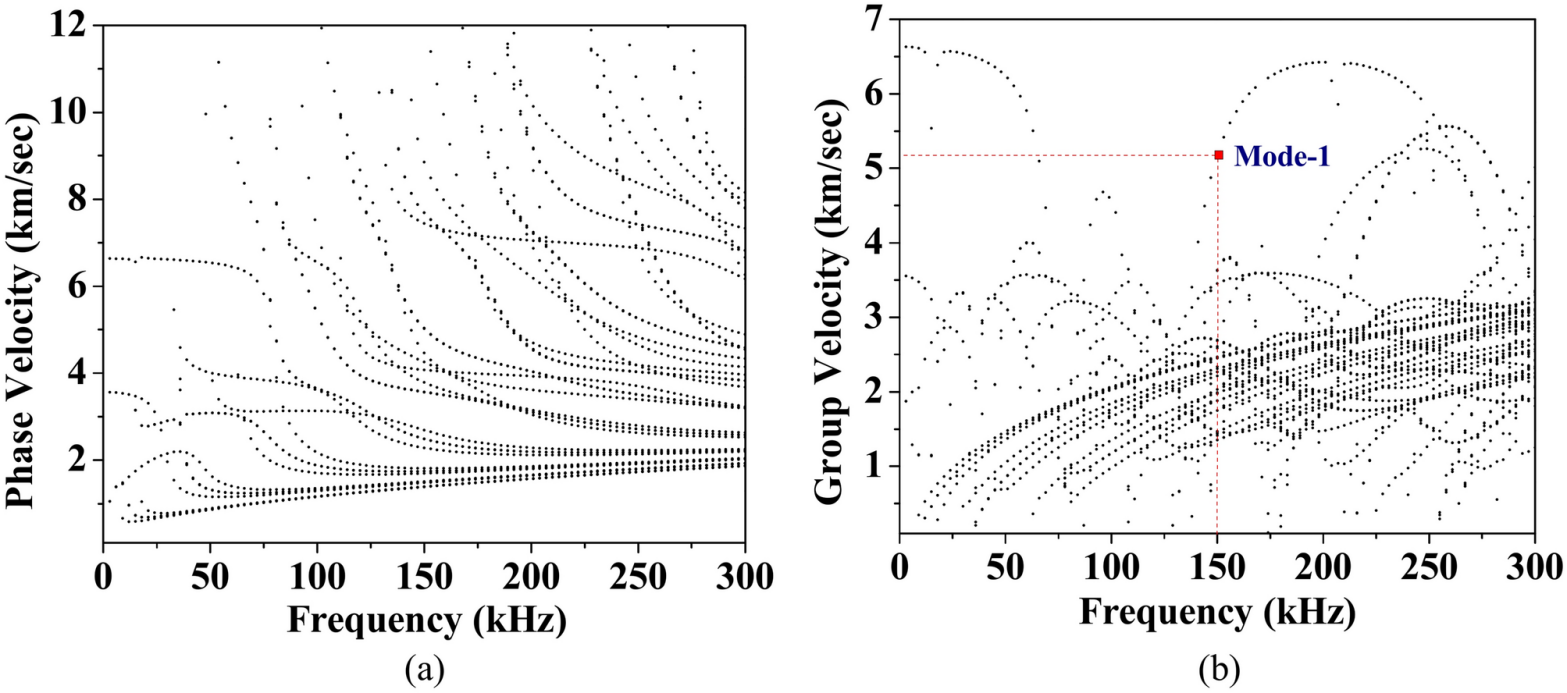
Dispersion curve of the hollow composite section (**a**) Phase velocity (**b**) Group velocity.

From Fig. 16, it can be noted that a large number of wave modes are generated at the lower excitation frequencies due to the complex geometry of the section. Additionally, it is challenging to distinguish the wave modes with group velocity below 3.5 km/sec due to the presence of numerous wave modes. However, in the comparison studies discussed in the previous section, it is noticed that one wave mode, ‘Mode-1’, is present in the waveform upto a time of flight of 120 µsec when excited with 5 cycles Hanning window at a central frequency of 150 kHz based on the group velocity that is calculated and compared with the developed dispersion curve, as shown in the group velocity plot as ‘Mode-1’.

**Fig. 17:**
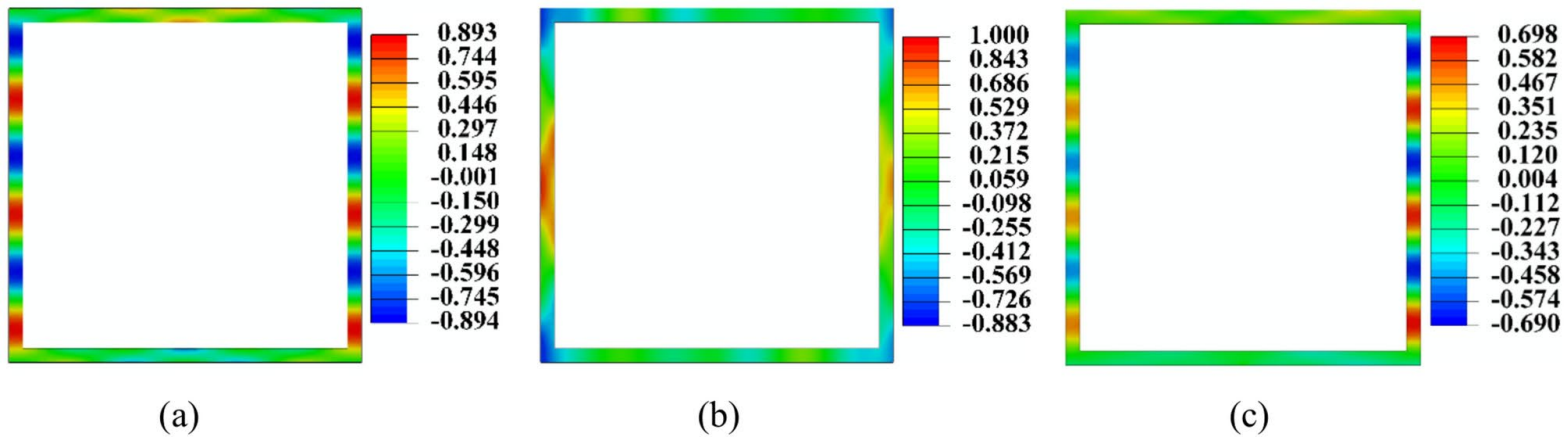
Cross-section displacement profile of the Mode-1 in terms of (**a**) *u*_*x*_*,* (**b**) *u*_*y*_, and (**c**) *u*_*z*_ field at an excitation frequency of 150 kHz.

Figure 17 presents the displacement profiles for the *u*_*x*_*, u*_*y*_, and *u*_*z*_ displacements corresponding to Mode-1 at a frequency of 150 kHz. The profiles reveal that none of the displacement fields exhibit symmetry or antisymmetry outlines with respect to any of the reference axes, which contrasts with the modes typically observed in plate or pipe sections. As a result, it is challenging to characterise this mode in a conventional manner, leading to its designation as ‘Mode-1’. Therefore, the developed dispersion curve aids in identifying the various modes present in the acquired waveform by comparing the velocity of the wavepacket calculated based on the time of arrival of the wavepacket in the acquired signal with the numerically obtained group velocity in the dispersion curve, which helps in understanding its variations for various parametric changes.

## Parametric behaviour studies

This section explores the detailed parametric studies are carried out on the HCM for different damage types and their parameters using the response obtained by the FE model. The impact of varying damage parameters are thoroughly studied through comparisons of various response features in subsequent subsections.

Studies focused on understanding the behaviour of the scattered guided wave under different damage parameters. Initially, a brief investigation is performed on the experimental response to assess how the excitation frequency affects the peak amplitude of the scattered response. After identifying the optimal excitation frequency,

### Effect of abrasion size on the guided wave behaviour

**Fig. 18:**
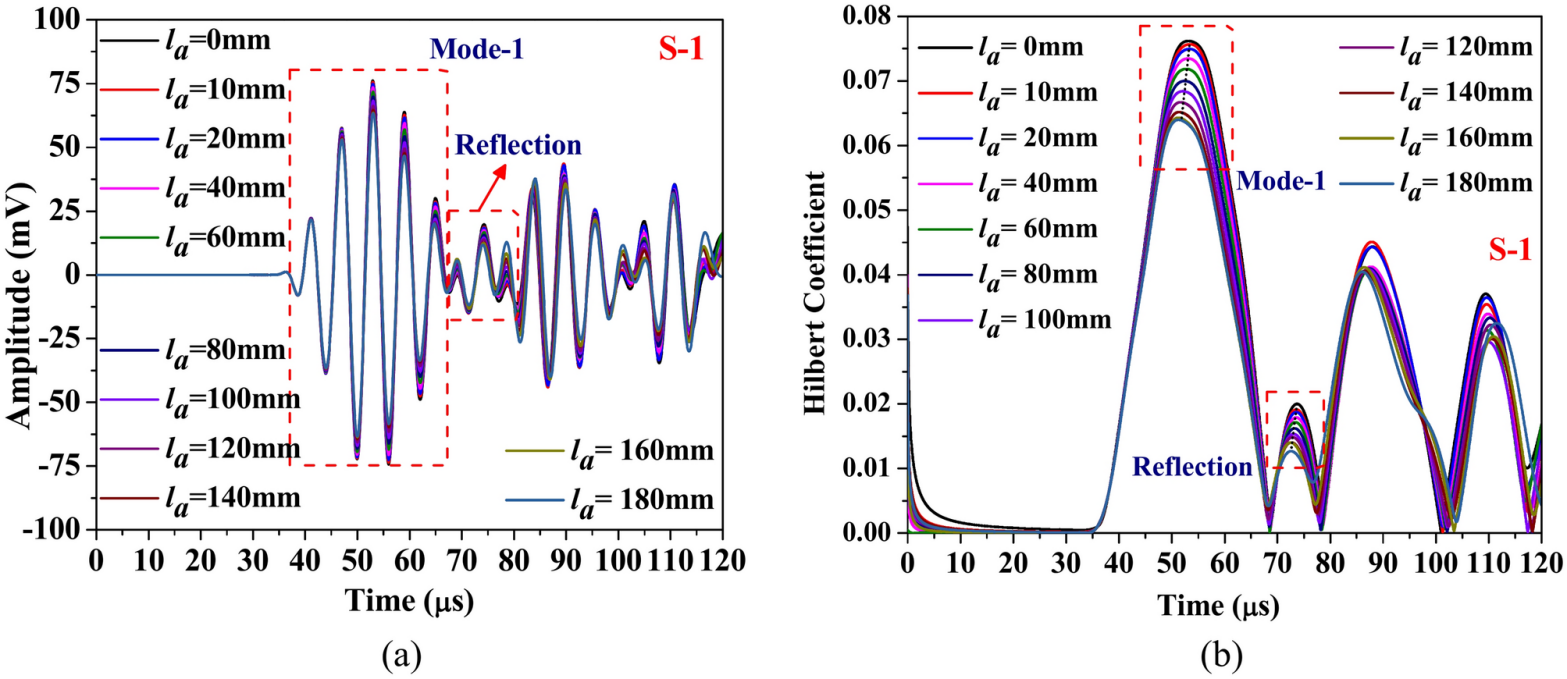
Influence of abrasion size on the (**a**) waveform and its (**b**) Hilbert transform envelope of the signal acquired through PZTs S-1.

**Fig. 19:**
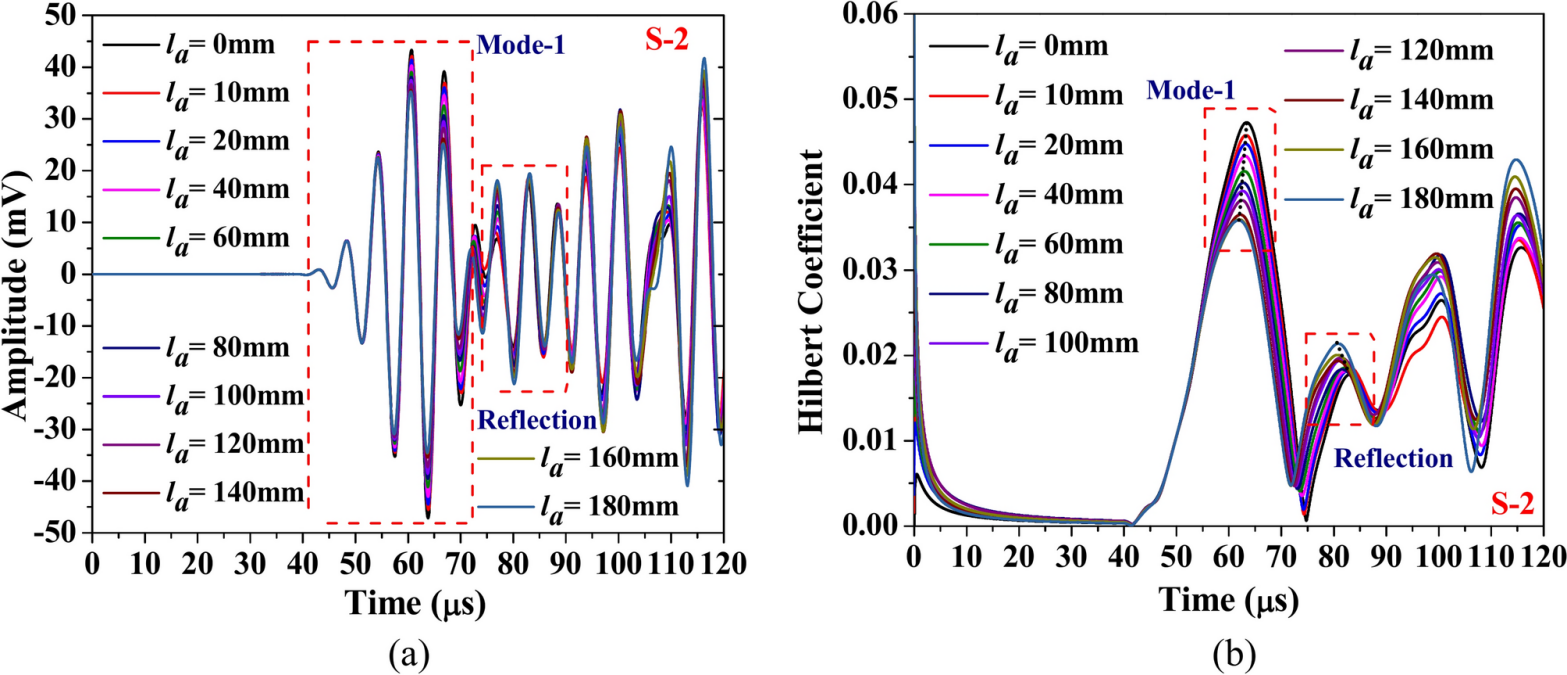
Influence of abrasion size on the (**a**) waveform and its (**b**) Hilbert transform envelope of the signal acquired through PZTs S-2.

This section discusses the study aimed at understanding the influence of abrasion length on the different guided waveform features of the scattered response obtained through the simulation. The study is conducted on the composite hollow section with through width abrasion and has an abrasion depth of 0.3 mm, located at an offset ‘*e*_*a*_’ of 190 mm from the edge. In the study, only the abrasion length is equally varied on either side. The waveform response and its Hilbert transform envelope, which is the absolute value of Hilbert transform, acquired through both PZTs are presented in Figs. 18 and 19. The figure shows that regardless of the PZT sensors, the amplitude of the Mode-1 wavepacket reduces with increasing abrasion length, accompanied by a slight advancement in arrival time, indicating a minor increase in group velocity. Conversely, the reflected wave packet in the response acquired through S-2 PZT propagates at a faster rate with the rise in the abrasion length. Notably, the amplitude of this reflected mode increases noticeably with abrasion length, which is contrary to the trend noticed in the reflected wavepacket in the response received at S1. This indicates that the behaviour of the reflected packet is highly sensitive to the receiver PZTs and has the potential to detect the change in the abrasion length.

**Fig. 20:**
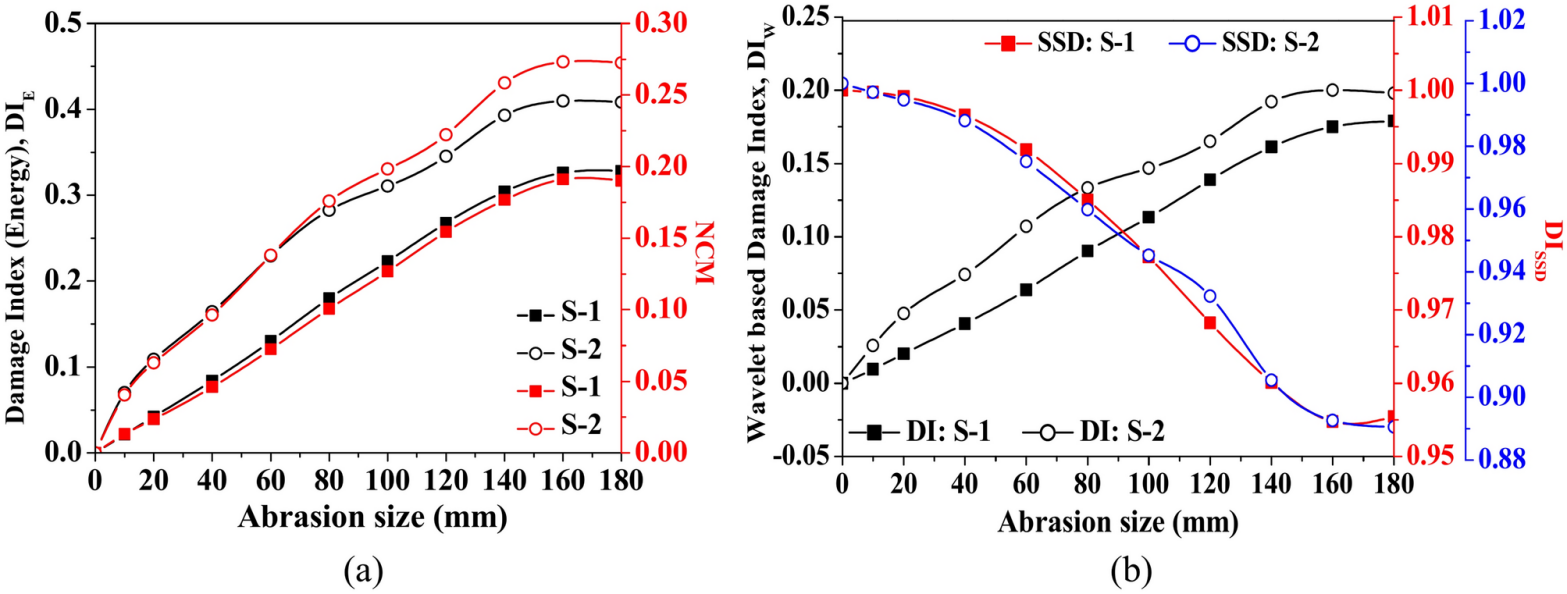
Influence of abrasion size on the various wave features based on the numerical response acquired through PZTs S-1 and S-2.

Furthermore, the impact of abrasion size on the damage index based on energy (DI_E_), wavelet coefficient DI_W_, the sum of square difference (DI_SSD_) and NCM are presented in Fig. 20 based on the signal captured through PZTs S-1 and S-2. Indices are estimated by considering the waveform within the time of arrival of the first two wavepackets. It is noticed that irrespective of the signal acquired through any PZTs, the damage indices based on energy and CWT coefficients (i.e., DI_E_ and DI_W_) and NCM initially enhance abruptly with the abrasion length upto *l*_*a*_ = 140 mm. Additional growth in the abrasion length beyond 140 mm has a minimal effect on these DI indices and NCM. Conversely, the index due to SSD reduces with abrasion length, which is contrary to the trend noticed in other damage indices and NCM. This trend in the wave features is basically due to the reduction in the wave amplitude of Mode-1 in the scattered response with the rise in the abrasion length.

**Fig. 21:**
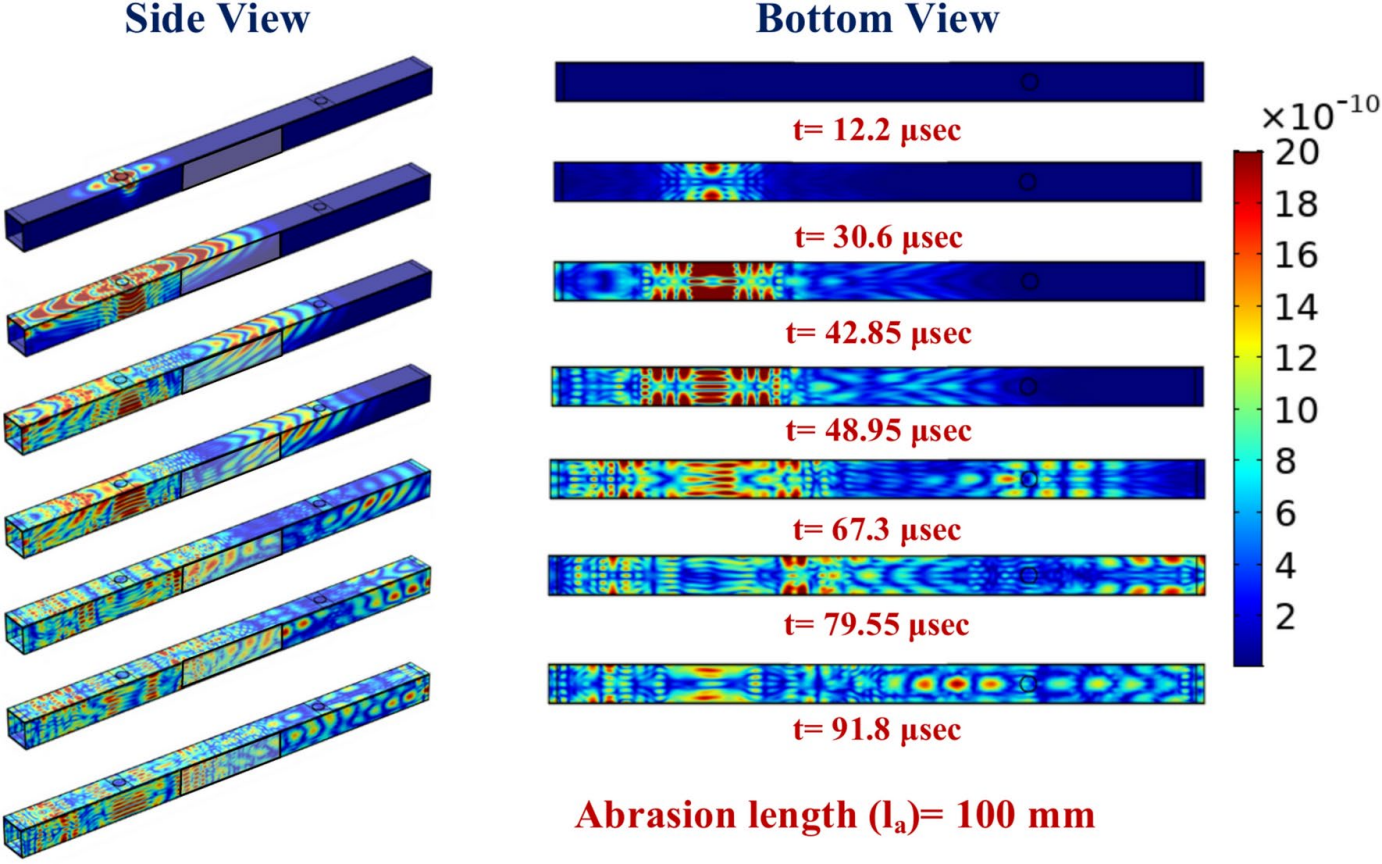
Displacement profile of the GW in the HCM with abrasion of 100 mm length at various time intervals.

Further, to gain a better insight into the response obtained in the complex hollow section, side view and bottom view plots illustrating the displacement profile around both mounted receiver PZTs and around the abrasion are shown in Fig. 21 at various time intervals. It is noticed that in the HCM, the wave propagates at a greater velocity along the section length and width due to the woven fabrics employed in the section. Later, with time, the wave travels along the receiver PZT S-1. However, in due course of time, because of the smaller sectional width compared to its length, multiple reflections occur from the edge and propagate along the PZT S-1, thereby mixing with the primary mode wave packet. Moreover, the presence of abrasion causes a minor wave reflection from the abrasion corners, which is difficult to distinguish in the displacement plot but can be understood by comparing the scattered waveform.

On the other side, due to greater speed in the width direction, the wave reaches the bottom surface from both the side faces and then the interference phenomenon of each wave packet occurs and further proceeds along the longitudinal direction towards PZT-S2. Later, the wave propagating along the diagonal direction from the actuator and receiver PZT S-2 through the abrasion region gets mixed up with the interference wave within a short time delay after the interface wave strikes the receiver PZT S-2. Further, multiple reflections from the section edges can be noticed. Thus, the study concludes that abrasion length significantly impacts guided wave features, with longer abrasions reducing Mode-1 wave amplitude and affecting damage indices like DI_E_, DI_W_, and NCM, which initially increase with length but stabilise beyond a certain point. This highlights the importance of abrasion length in affecting wave propagation characteristics, offering valuable insights for Structural Health Monitoring (SHM) systems in detecting and quantifying surface damage.

### Effect of abrasion position on the guided wave behaviour

**Fig. 22:**
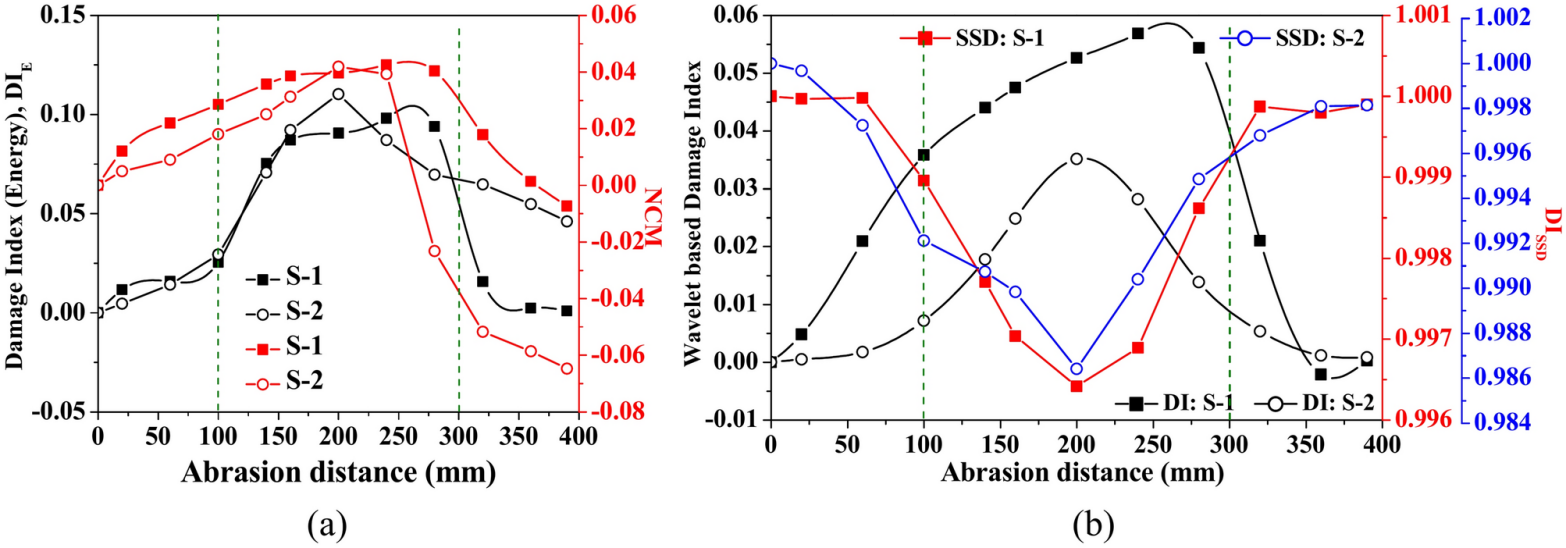
Influence of abrasion size on the various wave features based on the response acquired through PZTs S-1 and S-2.

The influence of abrasion position on the guided wave features is investigated in this section. The study is conducted on the HCM with a surface abrasion of length *l*_*a*_ = 40 mm and depth of 0.3 mm, with its position varied along the surface. The variation of different indices is illustrated in Fig. 22. Data is acquired from the numerical model by exciting the structure through a PZT mounted 100 mm from one edge, with the scattered wave received by a PZT positioned 300 mm from the edge, as indicated by the dotted line in Fig. 22. This setup aids in understanding the abrasion effect on the scattered wave feature within the actuator and receiver patches regime. The investigation shows that the value of damage indices based on the S-2 waveform’s energy and CWT coefficient rises marginally as the abrasion is positioned closer to the actuator-receiver PZT regime. However, a sharp rise in the magnitude of these indices are noticed when the damage is within the sensing region, i.e., from the actuator PZT to the mid-region of the actuator-receiver PZT regime. Further, as the abrasion is found to be closer to the receiver PZT, the magnitude of such indices drops. Similarly, irrespective of the acquired PZT response, the NCM value gradually increases with the position of abrasion up to the mid-region of the actuator-receiver PZT location, and then it reduces abruptly with the further rise in the offset. Conversely, a contrary behaviour is noticed, indicating the reduction in the DI_SSD_ value as the abrasion is positioned from the edge to the mid of the actuator-receiver position and then increases abruptly with a further rise in the distance. Notably, when the abrasion is within the direct response path zone of the actuator-receiver, an abrupt change in the DI_SSD_ value is observed, thereby indicating a significant variation in the signal amplitude. This scheme of the mounted PZTs can effectively detect the abrasion when induced within the direct response path region. The study highlights that the position of abrasion significantly influences guided wave features, with damage indices showing marked sensitivity when the abrasion is within the direct response path of the actuator-receiver PZT setup. This suggests that careful positioning of the abrasion within the sensing region enhances the effectiveness of guided wave-based Non-Destructive Testing (NDT) and Structural Health Monitoring (SHM) for detecting surface damage.

### Effect of abrasion depth on the guided wave behaviour

**Fig. 23:**
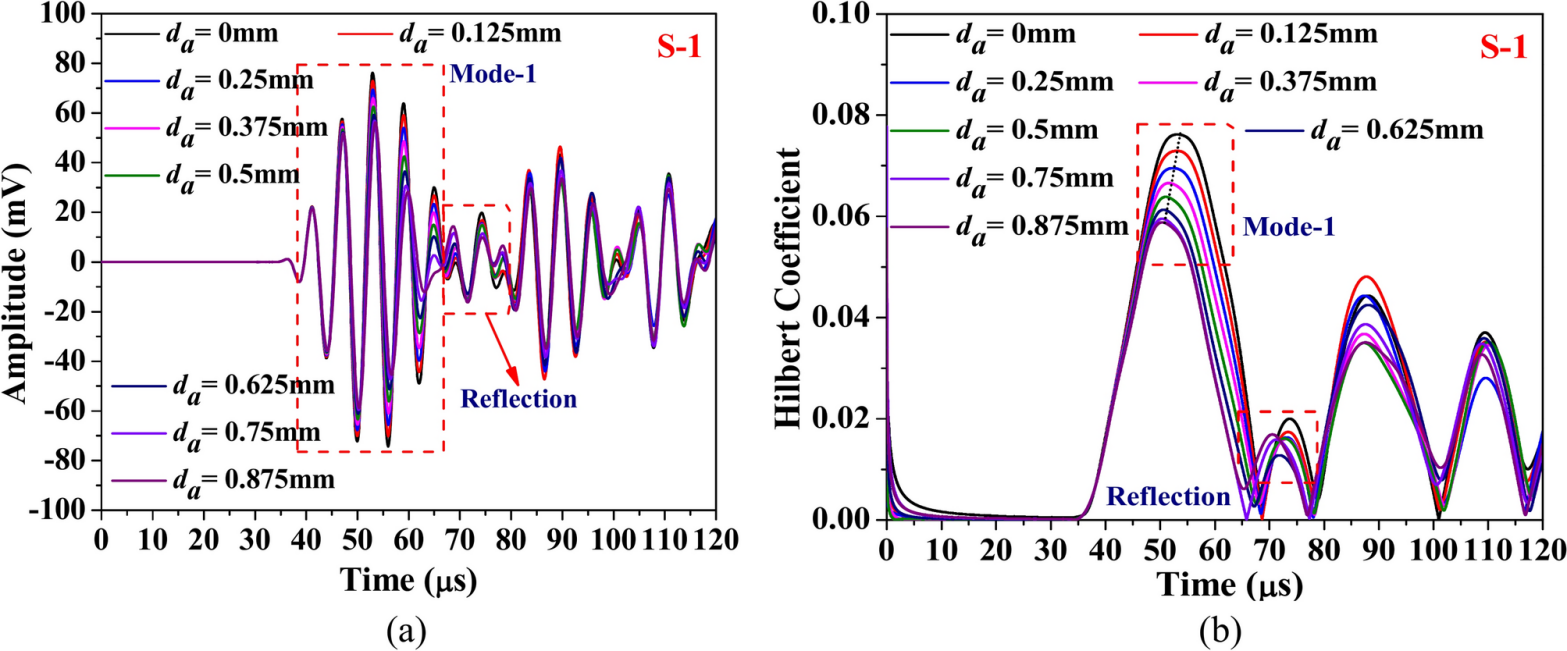
Influence of abrasion depth on the (**a**) waveform and its (**b**) Hilbert transform envelope of the signal acquired through PZTs S-1.

**Fig. 24:**
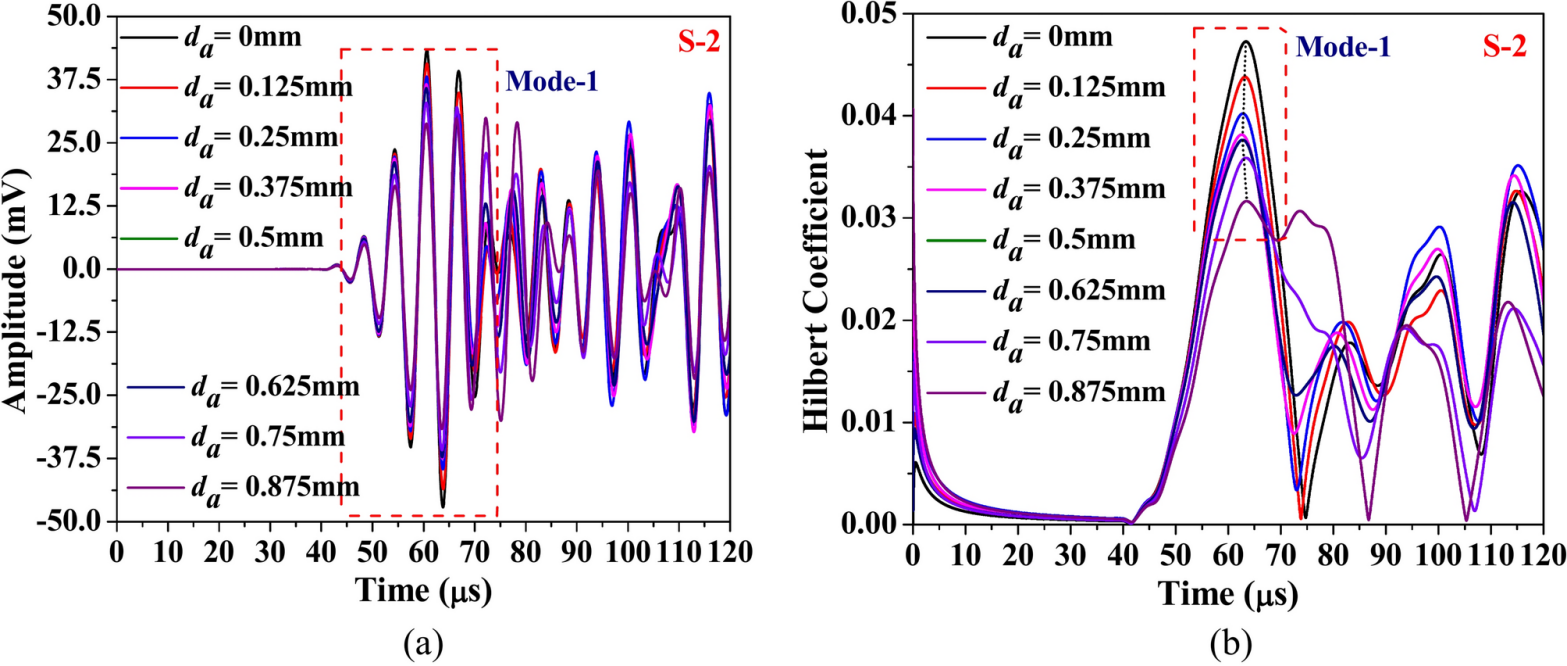
Influence of abrasion depth on the (**a**) waveform and its (**b**) Hilbert transform envelope of the signal acquired through PZTs S-2.

The investigation focuses on HCM with a centrally located through-width abrasion of 40 mm length, examining the effect of abrasion depth on the various wave features. The wave response and its Hilbert transform envelope of the response acquired through both the PZTs are presented in Figs. 23 and 24. It is observed that irrespective of PZTs response, as the abrasion depth increases, the amplitude of Mode-1 reduces with the rise along with the minor expedition in arrival time, indicating a slight enhancement in the group velocity. However, the amplitude of the reflected wavepacket initially reduces with abrasion depth ‘*d*_*a*_’ up to 0.625 mm, but a further rise in the depth enhances the wavemode amplitude.

**Fig. 25:**
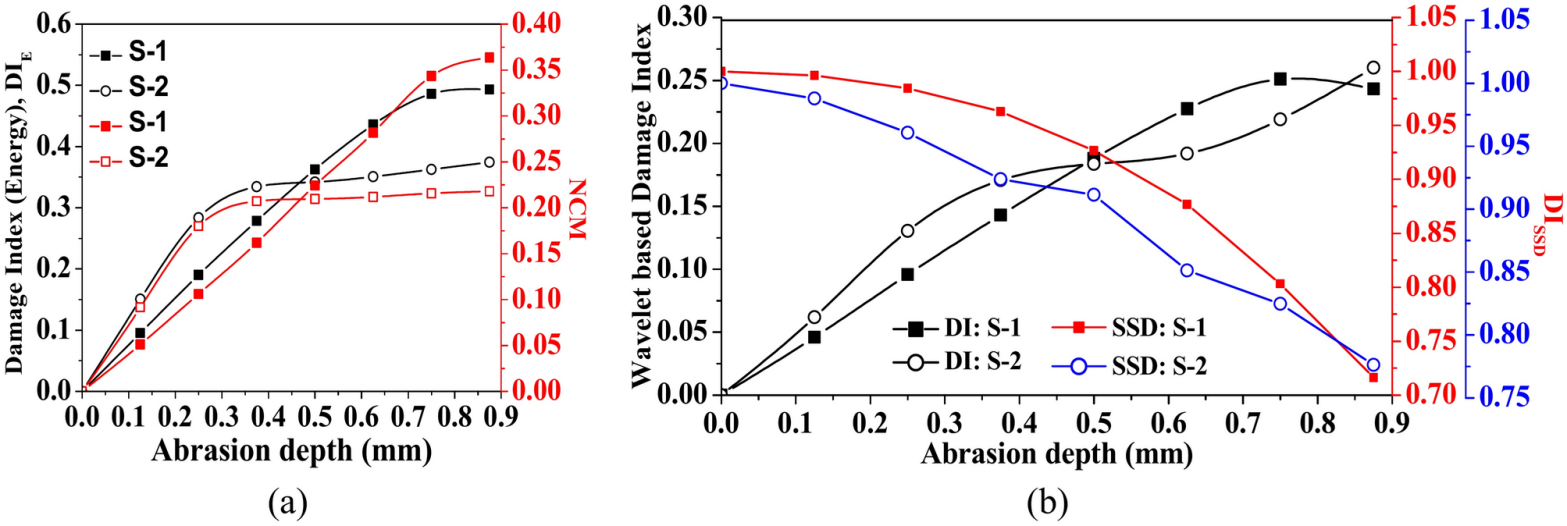
Influence of abrasion depth on the various wave features based on the response acquired through PZTs S-1 and S-2.

In addition, the variation of different indices with respect to abrasion depth is presented in Fig. 25. The study shows that the damage indices based on energy and wave coefficients (DI_E_ and DI_W_) enhance as the abrasion depth increases, regardless of the receiver PZT’s response. However, the NCM index’s magnitude abruptly enhances up to *d*_*a*_ = 0.375 mm, followed by a minimal increase with a further rise in depth. On the other side, the magnitude of index based on SSD reduces with the increase in the abrasion depth due to the reduction in the value amplitude, which is contrary to the trends noticed in the wavelet-based damage index. Moreover, the DI_SSD_ value based on S-2 response marginally reduces initially with abrasion depth up to *d*_*a*_ = 0.25 mm, and a further rise in abrasion depth leads to an abrupt reduction in DI_SSD_ magnitude. Therefore, the study concludes that abrasion depth significantly impacts wave parameters, with increasing depth generally leading to a reduction in Mode-1 amplitude and altered damage index behaviour, such as enhanced energy-based indices and a decrease in SSD-based indices, highlighting the substantial impact of abrasion on wave propagation and damage detection.

### Effect of hairline crack size on the guided wave behaviour

**Fig. 26:**
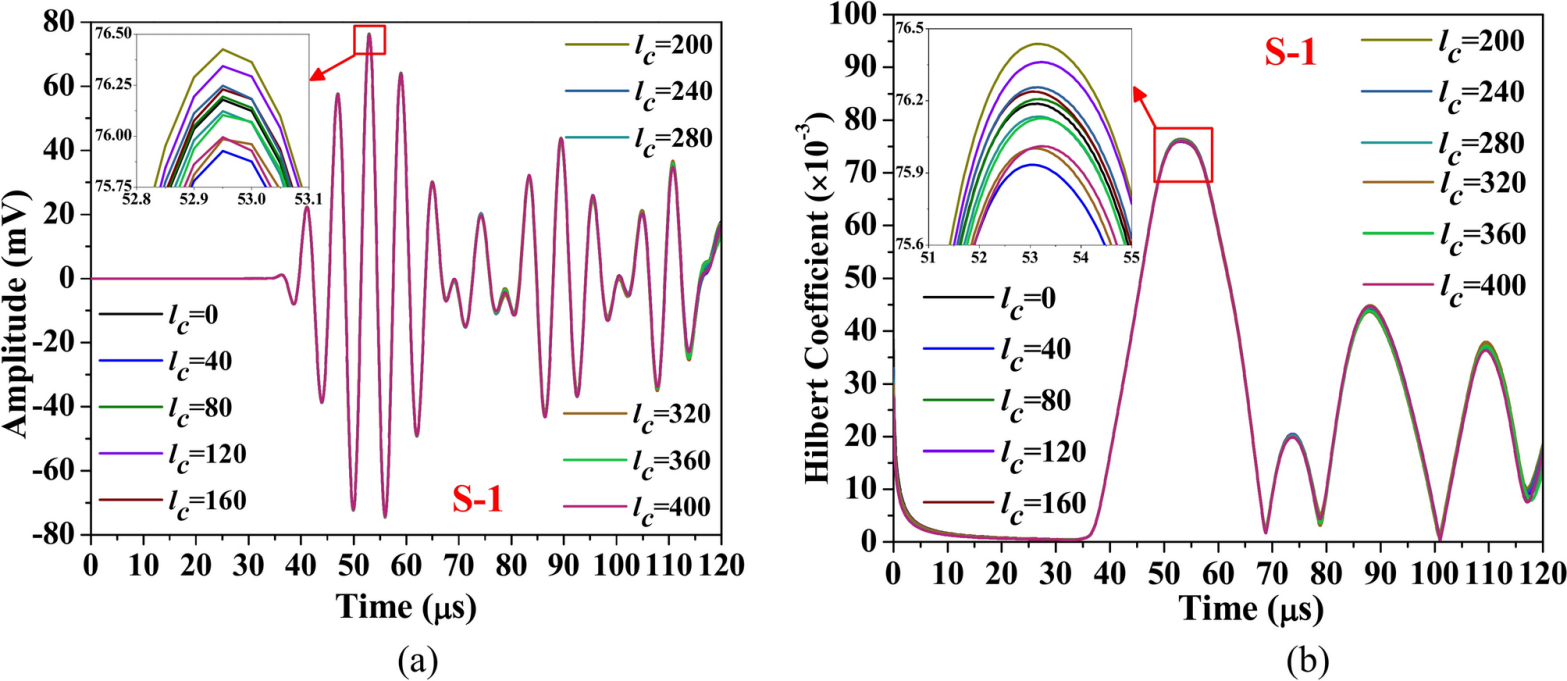
Influence of hairline crack size on the (**a**) waveform and its (**b**) Hilbert transform envelope of the signal acquired through PZTs S-1.

**Fig. 27:**
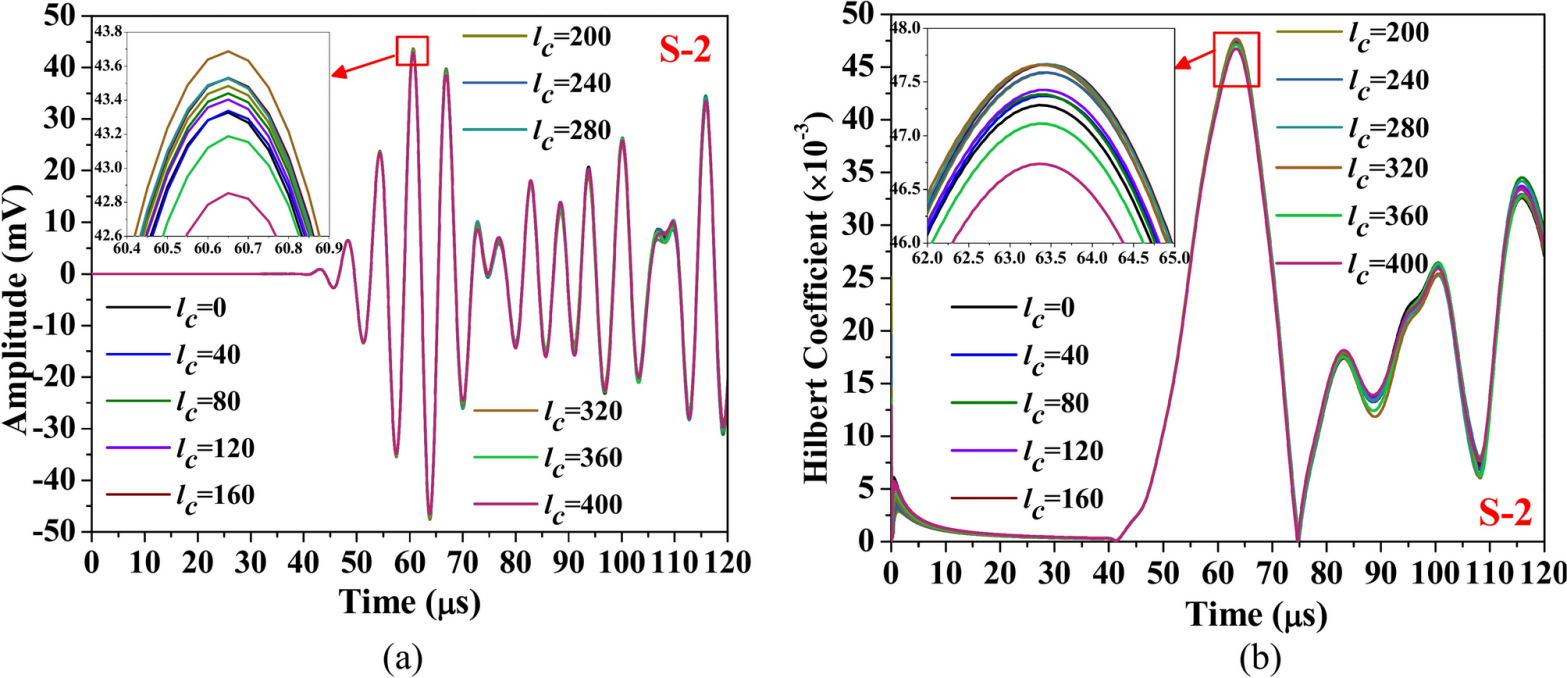
Influence of hairline crack size on the (**a**) waveform and its (**b**) Hilbert transform envelope of the signal acquired through PZTs S-2.

This portion of the study examines the effect of hairline crack size on various guided wave features. The investigation is conducted on HCM with a hairline crack of varying lengths initiating from the edge closer to the actuator PZT. The outcome of the present investigation in terms of waveform and its Hilbert transform envelope of the signal acquired through both reciever PZTs are illustrated in Figs. 26 and 27. It is noticed that the peak amplitude of Mode-1 in the response acquired through S-1 PZT initially enhances with the increase in the hairline crack up to a length of 200 mm, and a further increase in the crack length reduces the peak amplitude of the Mode-1 wave packet. Similarly, the peak amplitude of the response captured through PZT S-2 initially enhances with the rise in crack length up to 320 mm and then reduces as the crack continues to propagate. The reduction in peak amplitude with increasing crack length can be explained by various physical effects. As the crack length grows, guided waves interact more extensively with the damage, causing increased scattering and diffraction that diminish the transmitted signal’s amplitude. Additionally, longer cracks intensify mode conversion, redirecting wave energy into modes less effectively captured by the receiving sensor. The structural discontinuity introduced by larger cracks also amplifies energy attenuation during wave propagation through the damaged area.

**Fig. 28:**
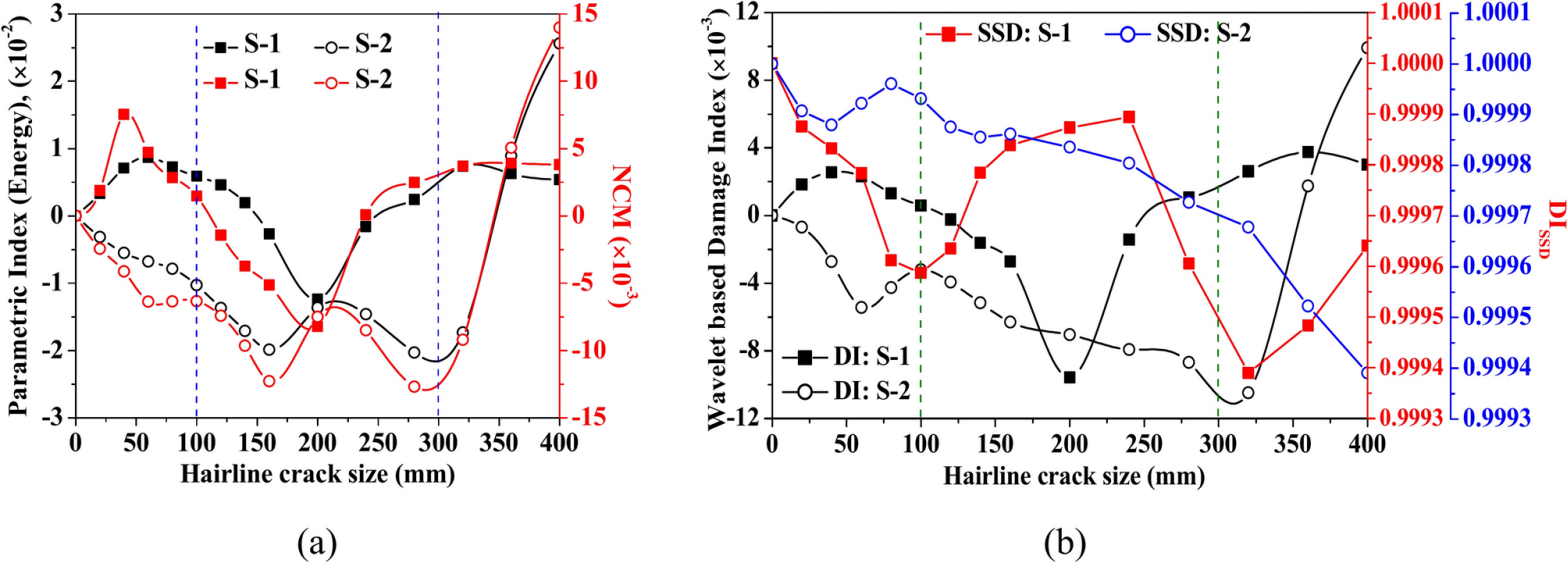
Influence of hairline crack length on the various wave features based on the response acquired through PZTs S-1 and S-2.

Along with considering the peak amplitude of the waveform, Fig. 28 presents the variation of damage indices due to various features and NCM magnitude under the variation of crack length. The dotted line at 100 mm and 200 mm in the figure indicates the position of mounted PZTs, highlighting the effect of crack size on the wave features relative to the attached PZTs. A contrary behaviour is noticed where the damage index due to energy and NCM value drops substantially up to a crack length of 200 mm and then enhances as the crack propagates further. Moreover, the magnitude of the damage index due to the wavelet coefficient similarly shows a sharp drop initially with the crack length, followed by an abrupt rise with further increases in length. The initial drop in the wavelet damage index is sensitive to the position of attached PZTs, through which response is obtained. Conversely, for the response obtained through S-1, contrary behaviour is noticed in the SSD damage index value to that of the wavelet damage index trend. This study infers that wave features are highly sensitive when the crack propagates in the zone between the actuator and receiver PZTs.

**Fig. 29:**
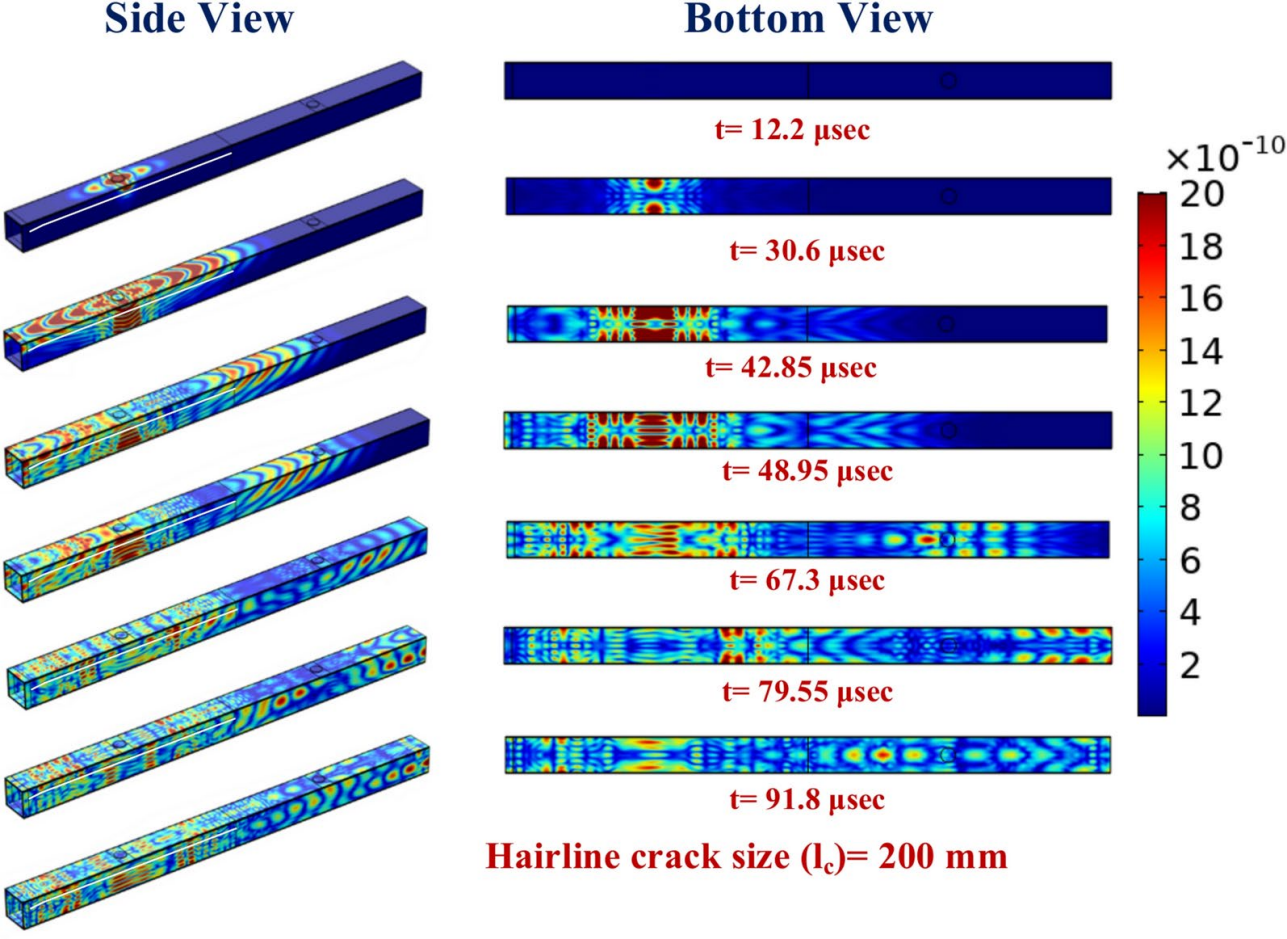
Displacement profile of the GW in the HCM with a hairline crack of 200 mm length from the one edge at various time intervals.

Further, the side view and bottom view plots presenting the displacement profile around both mounted receiver PZTs and around the hairline crack are illustrated in Fig. 29 at various time intervals to have better insight into the response obtained in the complex hollow section. From the plot, a similar trend is noticed as observed in the case of the hollow composite section with abrasion damage. However, a minimal change in the reflection pattern due to the hairline crack is noticed on the bottom face of the section beyond the time interval of 48.95 μs. In summary, the study highlights that wave features, including amplitude, group velocity, and damage indices, are highly sensitive to abrasion depth and crack length, particularly in the zone between actuator and receiver PZTs, offering valuable insights for enhanced damage detection. The findings underscore the importance of sensor placement and the intricate interplay between damage characteristics and wave responses for accurate diagnostics in complex hollow composite structures.

### Effect of hairline crack position on the guided wave behaviour

**Fig. 30:**
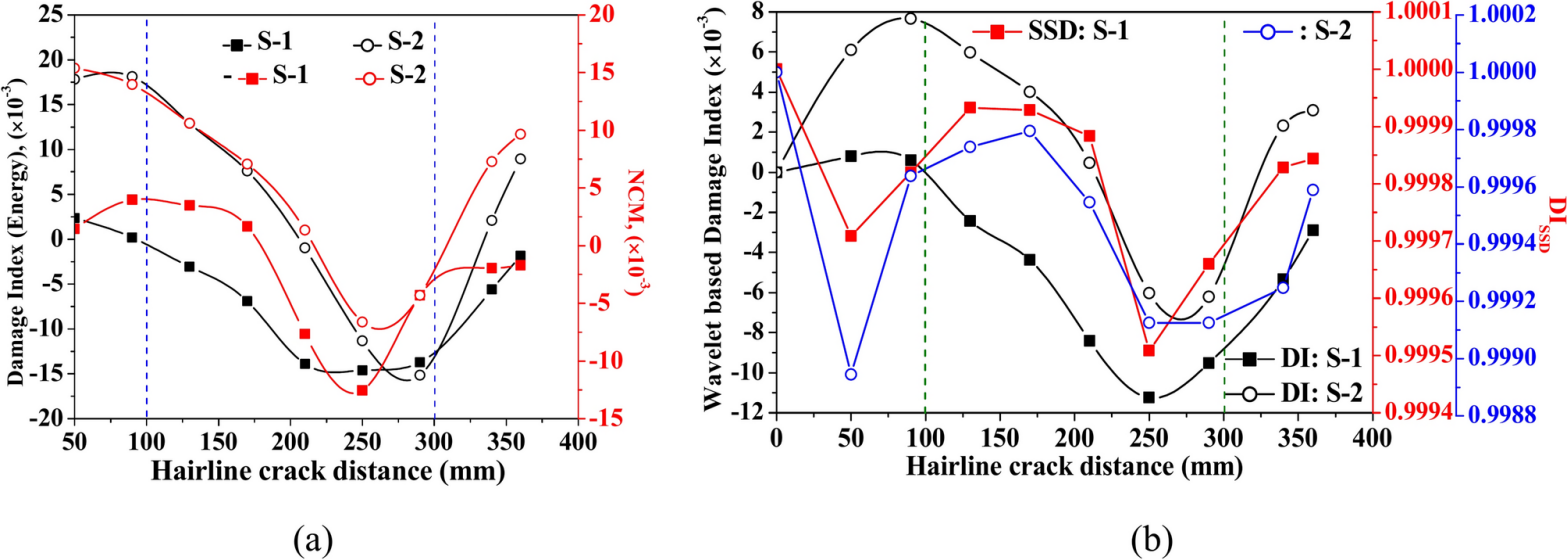
Influence of hairline crack position on the various wave features based on the response acquired through PZTs S-1 and S-2.

The following section includes the study investigating the influence of hairline crack position on the various wave features. The study examines a hairline crack of length 100 mm positioned at a distance of 12.5 mm from the actuator surface. The vertical dotted line at 100 mm and 300 mm indicates the actuator’s and receiver PZTs’ position to comprehend the variation due to a crack within this zone. From Fig. 30, it can be noticed that the value of the NCM and damage indices based on waveform energy and wavelet coefficient reduces with the increase in the crack offset up to the position closer to the mounted receiver PZTs. When the offset distance of the crack further increases beyond the actuator-receiver zone, the magnitude of these indices is enhanced. On the other side, the value of the SSD damage index significantly reduces as the crack is introduced at the edge. Furthermore, the amplitude of the DI_SSD_ index rises abruptly when the crack is present in the actuator-receiver zone upto distance of 250 mm, positioned from the actuator towards the receiver PZTs, and then increases significantly when the crack is present beyond the direct sensing zone of the PZTs. This highlights that the waveform’s amplitude increases substantially when the crack is present in the direct path-sensing zone between the PZTs, indicating that the wave features are highly affected by the cracks between the actuator and receiver PZTs. From this investigation, it is outlined that wave features, including damage indices and waveform amplitude, are significantly influenced by the position of a hairline crack, with maximum sensitivity observed when the crack lies within the actuator-receiver PZT zone. This emphasises the critical role of crack location in enhancing the accuracy of damage detection and diagnostics in NDT and SHM applications.

### Comparison between the abrasion and hairline crack damage in the hollow composite member

**Fig. 31:**
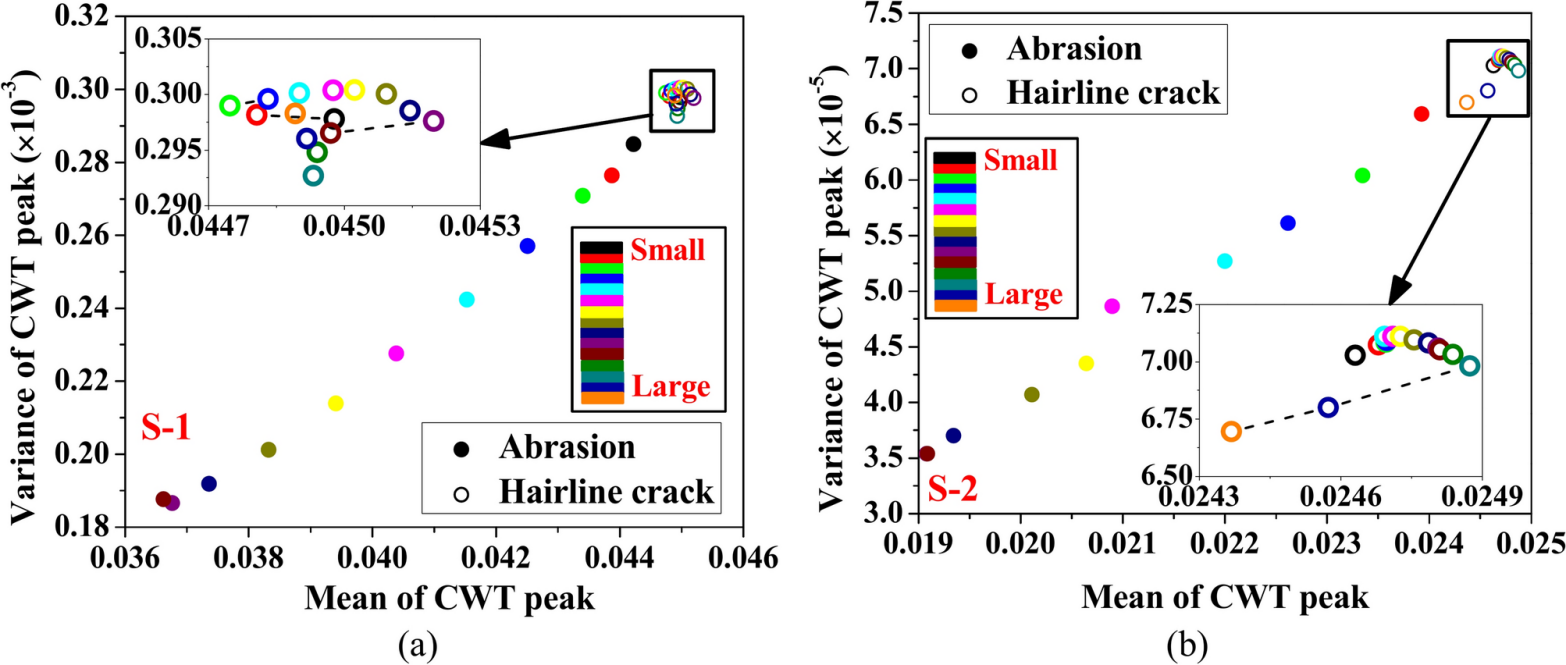
Comparison between the hairline crack and abrasion damage based on the wave features of the numerical response acquired through PZTs (**a**) S-1 and (**b**) S-2.

This investigation includes a comparison study to distinguish between the abrasion and hairline crack discontinuities considered in the current work. To achieve this, a plot of variance versus the mean value of the peak CWT coefficient of the response acquired numerically through mounted PZTs S-1 and S-2 over the frequency range of 90 kHz to 210 kHz is estimated for different lengths of abrasion and hairline damage, as described in Sect. 8.2 and 8.5. The variation of variance over the mean value of the CWT peak for various damage sizes is illustrated in Fig. 31. In the plot, the black colour point indicates a healthy state, while the brown colour point in the case of abrasion while the orange colour point for hairline crack highlights the wave feature corresponding to the maximum damage length. The variation in colour within the colour palette in the figure illustrates the increase in discontinuity length, ranging from 0 to 180 mm for abrasion and from 0 to 400 mm for hairline cracks. The plot clearly shows that for abrasion damage, the variance and mean of the peak CWT coefficient significantly reduce with the damage size, as illustrated by a linear drop in the wave features. In contrast, the variance and mean of the peak CWT coefficient exhibit minimal change for hairline crack damage. These features initially increase with the rise in the crack length and then reduce at higher crack size. These trends in the variation of mean versus variance of the peak CWT coefficient can aid one in identifying the damage types. Finally, it can be inferred that the abrasion damage substantially affects the wave features. In contrast, the hairline crack marginally influences the wave features of the response in the HCM. The study concludes that abrasion damage causes a significant reduction in wave features (variance and mean of the peak CWT coefficient), while hairline cracks have a minimal impact. This distinction enables precise identification of damage types in SHM, improving the reliability of structural diagnostics. Furthermore, for real-time applications, these feature variations can be utilised to pretrain using supervised Machine Learning algorithms, enabling it to predict both the type and size of the damage. The model can then use newly obtained wave features to make accurate predictions about the damage characteristics.

**Fig. 32:**
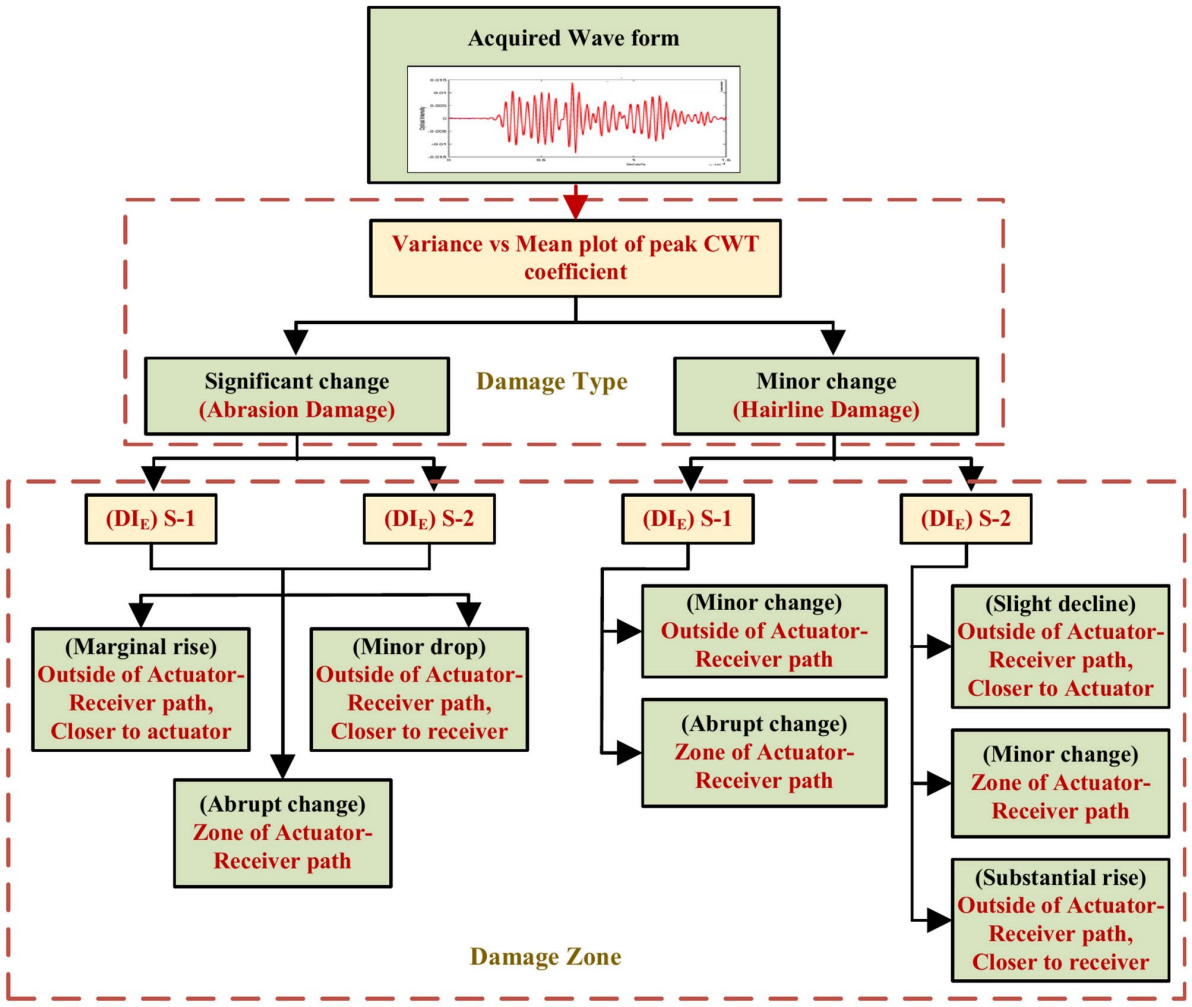
Flowchart illustrating the strategy to differentiate and locate the damage in the HCM.

A flowchart illustrating the strategy for identifying and locating damage zones is presented in Fig. 32. The proposed approach consists of two primary steps for damage identification and localization. As shown in the chart, the distinction between different types of damage, specifically abrasion and hairline cracks, is achieved by analyzing the plot of variance versus the mean of the CWT coefficient peaks. Significant changes in these statistical parameters are indicative of abrasion, whereas minor variations suggest the presence of hairline cracks. Furthermore, the damage location is determined based on the acquisition PZTs and by evaluating the Damage Index based on Energy content (DI_E_). The zonal positioning of the damage is inferred by considering the variations in DIE corresponding to the actuator-receiver pairs. Consequently, the proposed flowchart provides an effective means for distinguishing and localizing damage, facilitating practical implementation.

## Conclusions

This study investigates the impact of surface abrasion and hairline cracks on guided wave (GW) responses in thin-walled hollow composite members (HCM) using a hybrid theoretical–numerical–experimental framework. The key findings are as follows:

- The semi-analytical finite element (SAFE) dispersion analysis identifies multiple complex GW modes at lower frequencies, with Mode-1 wavepackets highlighting the substantial variability in the wave features due to damage in HCM.
- The signal statistical dispersion (SSD) damage index exhibits an inverse trend compared to energy-based and wavelet coefficient-based damage indices, offering a complementary diagnostic tool for real-world damage evaluation.
- GW features, including amplitude and group velocity, exhibit significant variations as damage size increases up to 160 mm, beyond which the changes become minimal.
- The wave response from the S-2 piezoelectric transducer (PZT) is highly sensitive to surface abrasion depth up to 0.25 mm, beyond which an increase in depth leads to negligible variations, informing practical limits for sensitivity in field applications.
- Sudden variations in GW features occur when abrasion or hairline cracks exist within the direct actuator-receiver zone, enabling localisation based on wave attenuation and feature changes.
- Hairline crack features become significant when the crack length or offset exceeds 300 mm from the edge, suggesting that the crack extends beyond the actuator-receiver zone.
- Comparative analysis shows that surface abrasion causes substantial changes in GW features and statistical parameters, while hairline cracks cause minimal changes, enabling field operators to differentiate damage types efficiently.

The study establishes that guided wave features and damage indices provide a robust basis for field-level identification and differentiation of damage types in hollow composite members, with notable sensitivity to surface abrasions and localised cracks within the actuator-receiver zone. These findings enhance NDT and SHM methodologies by enabling precise damage detection, localisation, and characterisation. The approach has the potential for extension to other composite damage types, such as delamination or impact damage. Future studies could focus on the use of nonlinear guided wave techniques for assessing breathing cracks or discontinuities in HCM, enhancing SHM applications for high-performance composite structures.

## Data Availability

The datasets used and/or analysed during this study are available from the corresponding author upon reasonable request.
